# Colonization With *Staphylococcus aureus* in Atopic Dermatitis Patients: Attempts to Reveal the Unknown

**DOI:** 10.3389/fmicb.2020.567090

**Published:** 2021-01-11

**Authors:** Patrycja Ogonowska, Yolanda Gilaberte, Wioletta Barańska-Rybak, Joanna Nakonieczna

**Affiliations:** ^1^Laboratory of Molecular Diagnostics, Intercollegiate Faculty of Biotechnology University of Gdańsk and Medical University of Gdańsk, Gdańsk, Poland; ^2^Department of Dermatology, University Hospital Miguel Servet, Zaragoza, Spain; ^3^Department of Dermatology, Venereology and Allergology, Medical University of Gdańsk, Gdańsk, Poland

**Keywords:** epidemiology of *S. aureus*, MRSA, staphylococcal enterotoxins, antistaphylococcal photodynamic treatment, virulence factor

## Abstract

Atopic dermatitis (AD) patients are massively colonized with *Staphylococcus aureus* (*S. aureus*) in lesional and non-lesional skin. A skin infection may become systemic if left untreated. Of interest, the incidence of multi-drug resistant *S. aureus* (MRSA) in AD patients is higher as compared to a healthy population, which makes treatment even more challenging. Information on the specific genetic background of *S. aureus* accompanying and/or causing AD flares would be of great importance in terms of possible treatment option development. In this review, we summarized the data on the prevalence of *S. aureus* in general in AD skin, and the prevalence of specific clones that might be associated with flares of eczema. We put our special interest in the presence and role of staphylococcal enterotoxins as important virulence factors in the epidemiology of AD-derived *S. aureus*. Also, we summarize the present and potentially useful future anti-staphylococcal treatment.

## Introduction

Atopic dermatitis (AD), also known as atopic eczema, is a chronic and relapsing inflammatory skin disorder. It may coexist with other atopic conditions: allergic rhinitis (hay fever), bronchial asthma and food allergy. AD mainly affects infants and young children. Nevertheless, it can persist or appear during puberty and adulthood. AD occurs commonly in 15–30% of children and 2–10% of adults worldwide (Silverberg, 2017).

**FIGURE 1:**
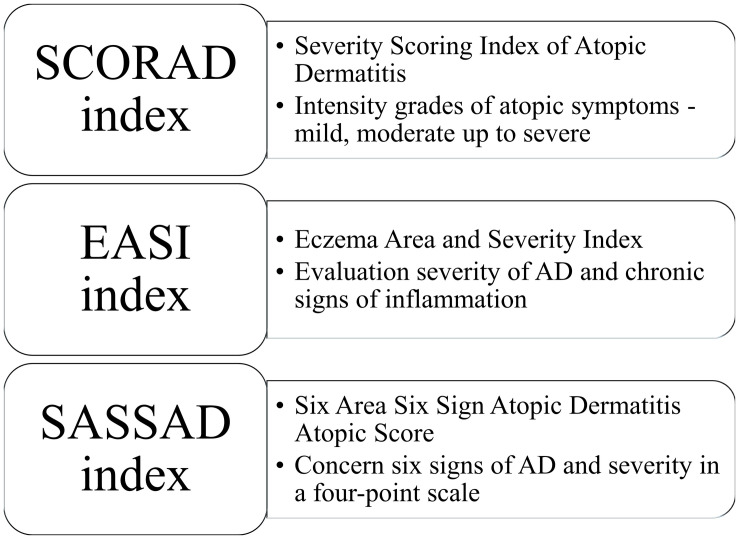
Various scoring systems for diagnosing atopic dermatitis.

In 1980, Hanifin and Rajka proposed criteria for diagnosing AD. According to the published guidelines, patients diagnosed with AD should present three or more basic features (e.g., pruritus, lichenification, atopic history) and three or more minor features (e.g., xerosis, early age of onset, food intolerance) (Hanifin and Rajka, 1980). Furthermore, various scoring systems have been established to measure disease severity. SCORAD (Severity Scoring Index of Atopic Dermatitis) evaluates the intensity of atopic signs in general in addition to the symptoms (pruritus and sleep) (Kunz et al., 1997), whereas EASI (Eczema Area and Severity Index) evaluates the severity of AD in four different parts of the body (head and neck, upper limbs, trunk and lower limbs) (Hanifin et al., 2001; Housman et al., 2002). SASSAD (Six Area Six Sign Atopic Dermatitis Atopic Score) less used includes six signs of AD (cracking, dryness, erythema, excoriation, exudation, and lichenification), their severity in a four-point scale (0—absent, 1—mild, 2—moderate and 3—severe) on the most following sites of different parts of the body (head and neck, arms, hands, trunk, legs, and feet) (Figure 1; Berth-Jones and Berth-Jones, 1996).

Currently, AD is considered a multifactorial skin disorder, with still not fully understood pathogenesis. The development of AD is a result of interactions between skin barrier defects and genetic, immunological and environmental factors (e.g., dust mite, tobacco smoke, soap, diet, air pollution, hygiene, stress) (Ring et al., 1992; Bonamonte et al., 2019). Patients suffering from atopic eczema revealed significantly reduced quality of life due to itching, which leads to sleep disturbances (Blome et al., 2016). The severe form of AD had a significant impact on the quality of life in adult patients compared to the mild and moderate types of AD (Chiesa Fuxench et al., 2019). Moreover, AD is a serious socio-economic problem in health care units because of the long treatment duration and financial costs (Carroll et al., 2005).

In addition to the long-term and burdensome treatment of AD patients, colonization by *Staphylococcus aureus* is another serious problem. *Staphylococcus aureus* is associated with the severity, pathogenesis and exacerbation of AD.

## Increased *Staphylococcus Aureus* Colonization Rate for AD Patients

The phenomenon of *S. aureus* colonization in AD patients has been known for a long time (Leyden and Marples, 1973; Hauser et al., 1985). Hauser et al. demonstrated higher *S. aureus* density, *S. aureus* fraction (SAF index) and total CFU/cm^2^ (CFU–colony forming units) in the lesional skin in AD patients than in the healthy control group (Hauser et al., 1985). In children suffering from AD, *S. aureus* colonization rate is higher than in the healthy group and affects 57–100% of children (Bunikowski et al., 2000; Arkwright et al., 2001; Lo et al., 2010; Pascolini et al., 2011). More than 40% of AD children are colonized both on the lesional skin and in the anterior nares (Pascolini et al., 2011). In children with AD, the carriage of the *S. aureus* strains in the anterior nares could be a potential source of recolonization (Patel et al., 2001). Results concerning *S. aureus* presence from AD children are summarized in Table 1.

**TABLE 1:** The distribution of *S. aureus* colonization and toxins production in **children** with atopic dermatitis.

References	Examined groups	Sites of isolation	Colonization *staphylococcus aureus*	Toxins production
Bunikowski et al. (2000) Germany	Children with AD (*n* = 74) Healthy control patients (*n* = 25)	Unaffected and eczematous skin lesions Neck Wrist Elbow Erosive eczematous lesions	AD patients 60 (81%) were *S. aureus* positive [including 40 (53%) toxigenic *S. aureus* strains] Healthy controls 5 (20%) were *S. aureus* positive [including 1 toxigenic *S. aureus* strain]	AD patients *sea*: 12, *seb*: 9, *sec*: 12, *sed*: 3, *tsst-1*: 9 Healthy controls *sea*: 1
Arkwright et al. (2001) United Kingdom	Children with AD (*n* = 28)	Eczematous lesions Nares	Each of the examined patients was colonized	Skin *sea*: 3 (11%), *seb*: 1 (4%), *sec*: 7 (25%), *sed*: 1 (4%), *see*: 0, *seg&sei*: 6 (21%), *seh*: 0, *tsst-1*: 3 (11%) Nose *sea*: 2 (7%), *seb*: 0, *sec*: 3 (11%), *sed*: 1 (4%), *see*: 0, *seg&sei*: 5 (17%), *seh*: 1 (4%), *tsst-1*: 2 (7%)
Lomholt et al. (2005) Denmark	Children with AD (*n* = 11)	Anterior nares Axillae Area of active eczema Perineum	No data	*sea:* 8 (29%), *seb:* 1 (4%), *sec:* 1 (4%), *sed:* 0 (0%), *tsst-1:* 0 (0%)
Lo et al. (2010) Taiwan	Children with AD (*n* = 133) Children with AD and SSTI (*n* = 20) Healthy controls (*n* = 490)	The anterior nares	Children with AD 67 isolates were positive for *S. aureus* (23 MRSA and 44 MSSA) Children with AD and SSTI 20 isolates were positive for *S. aureus* (12 MRSA and 8 MSSA) Healthy controls 170 isolates were positive for *S. aureus* (44 MRSA and 126 MSSA)	Results from molecular characteristics of 79 MRSA isolates from 643 children Children with AD *sea*: 1 (4%), *seb*: 20 (87%), *sec*: 2 (9%), *sed*: 0, *seg/sei*: 1 (4%), *seh*: 1 (4%), *tsst-1*: 2 (9%), Children with AD and SSTI *sea*: 0, *seb*: 12 (100%), *sec*: 0, *sed*: 0, *seg/sei*: 0, *seh*: 1 (8%), *tsst-1*: 0, Healthy controls *sea*: 3 (7%), *seb*: 28 (64%), *sec*: 11 (25%), *sed*: 1 (2%), *seg/sei*: 11 (25%), *seh*: 0, *tsst-1*: 7 (16%)
Pascolini et al. (2011) Italy	Children with AD (n = 117) Healthy controls (*n* = 90)	Skin lesions Normal skin areas Nares	Children with AD 66 patients (57%) - lesional skin and nares: 47 (40.2%) - nares: 19 (16.2%) - uninvolved skin: 4 (3.4%) Healthy children 18 patients (20%) - nares: 18 - uninvolved skin: 0	Enterotoxins 71 positive among 90 *S. aureus* strains *Tsst-1* 40 positive among 90 *S. aureus* strains
Park et al. (2013) Korea	Infants with AD (n = 188) Children with AD (*n* = 267) Control group— patients with urticaria (*n* = 247)	Skin lesions (acute and chronic)	Infants - acute lesion: 50% (18/36) - chronic lesion: 18.5% (28/151) Children - acute lesion: 80% (44/55) - chronic lesion: 41.8% (90/215)	No data
Gilaberte et al. (2015) Spain	Children with AD (*n* = 114)	Clinically uninfected lesional skin (antecubital or popliteal areas) Nares	Skin: 32/113 (28.3%) Nares: 20/85 (23.5%) All *S. aureus* strains were MSSA except one MRSA isolated from the skin	Skin and nasal isolates *seb:* 1 (2.5%), *sec*: 2 (5%), *tsst-1*: 22 (55%) Skin isolates *seb:* 0, *sec:* 2 (7.7%), *tsst-1:* 13 (50%)
Abad et al. (2019) Brazil	Children with AD (*n* = 117)—2 months–14 years old	Nasal swabs	97/117 of patients (82.90%) were colonized with *S. aureus* - 26/97 (22.22%) MRSA - 71/97 (60.68%) MSSA	No data

Regarding *S. aureus* colonization in adult patients, 54–100% (Breuer et al., 2002; Tomi et al., 2005; Gong et al., 2006; Kim et al., 2009; Na et al., 2012; Rojo et al., 2014; Clausen et al., 2017, 2019) suffering from AD were colonized by this species. *S. aureus* isolates formed a reservoir in the nose in AD patients. It can be diffused by autotransmission on the skin area (Breuer et al., 2002). The most colonized site is lesional skin (56–96.2%) (Matsui et al., 2000; Park et al., 2016; Totté et al., 2016; Alsterholm et al., 2017), nose (46.1–64.1%) (Na et al., 2012; Park et al., 2016; Totté et al., 2016; Clausen et al., 2017), and non-lesional skin (28–39%) (Matsui et al., 2000; Totté et al., 2016; Clausen et al., 2017). Several studies demonstrated that 65–77.3% (Breuer et al., 2002; Tomi et al., 2005; Na et al., 2012) of AD patients were colonized both in the anterior nares and on the skin, whereas only 10.2% of healthy control subjects were colonized on the skin (Matsui et al., 2000). Alsterholm et al. (2017) found that 55% of the AD patients were persistent carriers of *S. aureus*. Moreover, persistent *S. aureus* carriers had a higher SCORAD than intermittent carriers or non-carriers. It turns out that not only the nose but also the skin could be an important reservoir of *S. aureus* in AD patients (Alsterholm et al., 2017). Frequent recolonization by *S. aureus* between nose and skin was observed, which can contribute to the severity of AD (Chiu et al., 2009). Results concerning the distribution of *S. aureus* in adults are summarized in Table 2.

**TABLE 2:** The distribution of *S. aureus* colonization and toxins production in **adults** with atopic dermatitis.

References	Examined groups	Sites of isolation	Colonization of *staphylococcus aureus*	Toxins production
Zollner et al. (2000) Germany	AD patients (*n* = 33) Atopic controls (*n* = 21) Healthy controls (*n* = 50)	AD patients: mucous membranes (nose and throat), involved skin Healthy controls: healthy skin of the elbows	AD patients: 23/33 (69.70%) Atopic controls: 9/21 (42.86%) Healthy controls: 15/50 (30%)	AD patients 13/23 (57%) isolates produced SEs *seb* (5/13, 38%), *sec* (1/13, 8%), *sed* (1/13, 8%), *tsst-1* (3/13, 23%) Atopic controls 3/9 (33%) isolates produced SEs *sea* (2/3, 66%), *seb* (1/3, 33%), *tsst-1* (1/3, 33%) Healthy controls 5/15 (33%) isolates produced SEs *sea* (1/5, 20%), *seb* (1/5, 20%), *tsst-1* (3/5, 60%)
Breuer et al. (2002) Germany	Patients with AD–adults (*n* = 66)	Skin Anterior nares	62 of the 66 patients (94%) - skin(+), nose(+): 51 (77.3%) - skin(+), nose(−): 7 (10.6%) - skin(−). nose(+): 4 (6.1%) - skin(−), nose(−): 4 (6.1%)	Cutaneous and nasal isolates from 32 patients were included in the study. 10 (31%) of the patients were colonized with toxigenic *S. aureus* Skin *sea* = sed > *seb* > *sec* = *tsst-1* Nose *sed* > *seb* > *sea* = *sec* *tsst-1* was not detected
Schlievert et al. (2008) United States	Group 1 isolates Patients with steroid-resistant atopic dermatitis (*n* = 78) Group 2 isolates Healthy women vaginas (*n* = 30) Group 3 isolates Patients with atopic dermatitis (*n* = 22)	Group 1 isolates: 4 the most affected eczematous lesions	No data	Group 1 *sea*: 37 (47%), *seb*: 33 (42%), *sec*: 23 (29%), *sed*: 38 (49%), *see*: 33 (42%), *sei*: 38 (49%), *tsst-1*: 27 (35%) Group 2 *sea*: 8 (27%), *seb*: 3 (10%), *sec*: 9 (30%), *sed*: 4 (13%), *see*: 6 (20%), *sei*: 10 (33%), *tsst-1*: 12 (40%) Group 3 *sea*: 4 (18%), *seb*: 2 (9%), *sec*: 2 (9%), *sed*: 1 (4.5%), *see*: 6 (27%), *sei*: 10 (45%), *tsst-1*: 11 (50%)
Kim et al. (2009) Korea	Adolescent or adult patients with AD (*n* = 42)	The eczematous lesions: Lateral neck Forearm Abdomen Popliteal area	35 of the 42 patients (83.3%)	*sea*: 35 (97.2%), *seb*: 1 (2.8%), *sec*: 0, *sed*: 5 (13.9%), *see*: 0, *tsst-1*: 35 (97.2%)
Park et al. (2013) Korea	Adults with AD (*n* = 232) Control group—patients with urticaria (*n* = 247)	Skin lesions (acute and chronic)	Adults - acute lesions: 87.5% (35/40) - chronic lesions: 48.9% (93/190)	No data
Alsterholm et al. (2017) Sweden	Patients with AD (*n* = 21)	Lesional skin Anterior nares Perineum Tonsils Non-lesional skin	Lesional skin: 57–65% Non-lesional skin: 53–71% Anterior nares: 53–67% Tonsils: 24–30% Perineum: 32–55%	No data
Clausen et al. (2019) Denmark	AD patients (*n* = 63)	Lesional skin Non-lesional skin Nose	34 of patients (54%): - lesional skin: 33% - non-lesional skin: 10% - nose: 41%	No data

High *S. aureus* colonization rate is observed in both groups, children and adults. Colonization rate increases with the severity of the AD, and it acts as an aggravating factor exacerbating inflammation (Breuer et al., 2002). Also, colonization with *S. aureus* in AD patients could be a potent risk of various invasive infections, e.g., bacteremia, septic shock, osteomyelitis, necrotizing pneumonia, or septic arthritis (Patel and Jahnke, 2015).

## MRSA vs. MSSA Distribution in Atopic Dermatitis

Among *S. aureus* isolates, methicillin-resistant *S. aureus* (MRSA) constitutes an important and significant group that requires particular concern. MRSA is a group of strains that are resistant to multiple β-lactam antibiotics (cephalosporins, carbapenems, monobactams, and penicillins). This phenotype results in limited treatment options, including for skin infections (Rangel and Paller, 2018). It has been demonstrated that among *S. aureus* strains colonizing AD patients, the percentage of MRSA is 4–13 times higher than in a healthy population (Suh et al., 2008; Lo et al., 2010).

Currently, three profiles of MRSA are distinguished: hospital-associated (HA-MRSA), community-associated (CA-MRSA), and livestock-associated MRSA (LA-MRSA). Initially, epidemiological investigations indicated that MRSA infections related only to hospitalized patients (HA-MRSA). However, later it turned out that MRSA can also be isolated from infected people that have not been exposed to healthcare-related risks (CA-MRSA). The first outbreak of CA-MRSA was described in 1981 in the United States (Saravolatz et al., 1982) and nowadays is associated with skin and soft tissue infections (King et al., 2006; Chung et al., 2008). The skin of patients with AD could be a favorable reservoir for CA-MRSA. In the United States, 18.3% of AD patients are colonized with CA-MRSA (Chung et al., 2008). Colonization with MRSA constitutes the best-known risk factor for developing infection and MRSA can be easily transferred via direct skin-to-skin contact in the public settings, e.g., gyms, thus spreading the bacteria further. Reported in recent years, LA-MRSA is of animal origin, but it has also been detected in humans (van Cleef et al., 2011). It typically causes skin and soft tissue infections (SSTI) as well as more severe infections, similar to HA- and CA-MRSA. Some epidemiological data on the prevalence of LA-MRSA skin and soft tissue infections account for 15% of all MRSA SSTI infections in the community (Butaye et al., 2016). However, similar data in the AD population are not currently available.

Nasal carriage of *S. aureus* plays a vital role in the epidemiology and pathogenesis of AD disease. Nevertheless, the distribution of MRSA strains among AD patients is still divergent in the worldwide population. Among *S. aureus*-positive swabs from the anterior nares of AD patients, 34% were MRSA, in contrast to 26% from healthy children (Lo et al., 2010). In another study carried out in Brazilian AD children, 22.22% of *S. aureus* isolated strains were MRSA. On the other hand, when Korean AD children skin lesions were screened, 18.4% *S. aureus* strains were MRSA. Finally, among the isolates from AD children from Italy, only 7.9% were MRSA (12.8% from the skin lesion and 4.5% from the nose) (Chung et al., 2008; Pascolini et al., 2011). Moreover, they indicated that children with AD who had contact with wounds and pus at home or with persons colonized by MRSA had an increased risk of acquisition MRSA. In the case of adults with AD, the overall distribution of MRSA isolates seems to be much lower (Kim et al., 2009) or even absent (Rojo et al., 2014), as compared to the children in AD population.

Studies mentioned above indicate that the distribution of MRSA isolates among AD patients reflects an increased prevalence of MRSA in the AD population as compared to healthy ones, in particular in children. Notably, among patients expressing a severe type of AD, a higher risk of MRSA acquisition with time was reported in comparison to patients with a mild or moderate AD type (Abad et al., 2019).

## Factors Predisposing to the *S. Aureus* Colonization

**FIGURE 2:**
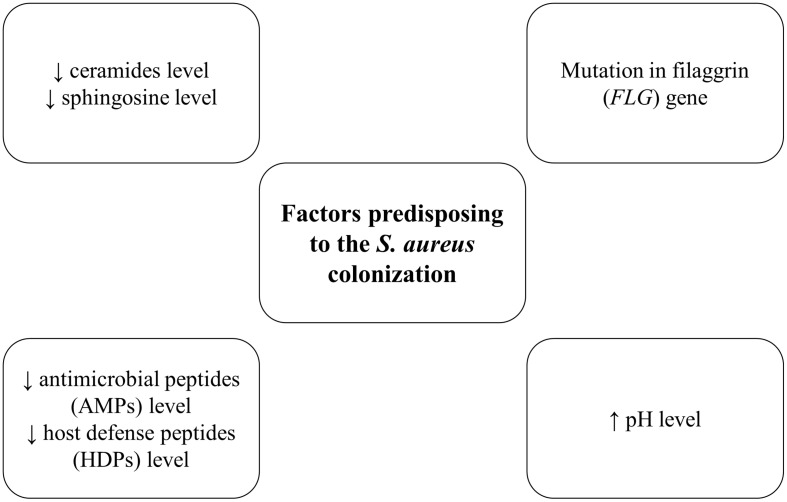
Factors predisposing to the *S. aureus* colonization.

One of the main factors predisposing to the *S. aureus* colonization are changes in the composition of lipids and fatty acids in the skin. In the epidermis (especially in the *stratum corneum*) significantly lower level of ceramides and higher amount of cholesterol was observed (Figure 2; Murata et al., 1996; Di Nardo et al., 1998). The reduction of skin lipids level could explain the role of these components in maintaining the hydration of the skin (Coderch et al., 2003). Similarly, ceramides and sphingosine levels are reduced in the stratum corneum of AD patients, which may favor *S. aureus* colonization. It was shown that sphingosine reveals the antimicrobial effect against *S. aureus* (Arikawa et al., 2002). Furthermore, *S. aureus* that colonized patients with AD produced an enzyme—ceramidase (Ohnishi et al., 1999). Since ceramides play a crucial role in the water-retaining in the *stratum corneum*, ceramidases action lead to the deficiency of ceramides molecules, which is associated with increasing trans-epidermal water loss and characteristic dry, cracked skin in patients with AD (Arikawa et al., 2002).

The level of antimicrobial peptides (AMPs) and host defense peptides (HDPs), produced by keratinocytes such as dermicidin, human β-defensins and cathelicidin–LL-37 are markedly reduced in AD skin, which also conduces to *S. aureus* colonization and infection (Ong et al., 2002; Roll et al., 2004). These peptides efficiently inhibit *S. aureus* growth (Niyonsaba et al., 2017). Th2 cytokines IL-4, IL-13, and IL-31, which are overexpressed in AD patients, inhibited the expression of the human β-defensins genes (hBD-2 and hBD-3). It is probably one of the factors that contribute to the proliferation of *S. aureus*, disturbance of microbiota composition and implication in the AD pathogenesis (Kanda and Watanabe, 2012).

Filaggrin (FLG) is an epidermal protein which is a part of the stratum corneum, the main barrier of the skin. Filaggrin is responsible for hydration, maintaining epidermal homeostasis, creating chemical, and structural barrier function (O’Regan and Irvine, 2008). The primary function is bonding keratin cytoskeleton in the process of keratinocytes maturation in the skin layer (Candi et al., 2005; Brown and McLean, 2012). Filaggrin can act as a scaffold for the connection of the lipids layers (O’Regan et al., 2008). As a result of filaggrin breakdown, pyrrolidone carboxylic acid (PCA) and urocanic acid (UCA) are formed, which are the composition of natural moisturizing factor (NMF) (Rawlings et al., 1994; O’Regan et al., 2008). This factor plays a crucial role in maintaining hydration of the *stratum corneum* and the appropriate pH of the skin (Rawlings et al., 1994; O’Regan et al., 2008). Nowadays, most studies proved that filaggrin loss-of-function mutations play an important role in the aggravation process in patients with AD (Brown and McLean, 2012). Reduced levels of filaggrin cause skin inflammation, resulting from the increasing penetration of allergens or irritants (Gruber et al., 2011). Furthermore, the levels of filaggrin and NMF are significantly decreased in AD patients (Brown and McLean, 2012). Also, PCA and UCA, filaggrin breakdown products have been *in vitro* shown to impact *S. aureus* cell density and growth rate (Miajlovic et al., 2010). Notably, in patients with a mutation in the filaggrin gene (*FLG*) increased *S. aureus* colonization was showed as compared to wild-type patients (Clausen et al., 2017).

In the skin, pH level plays a key role in maintaining the proper barrier function in the epidermis, protection against pathogens and control of the process of desquamation. The pH level of healthy skin is slightly acidic (4.0–6.0). Fatty acids–products of the phospholipid hydrolysis in sebum and sweat, maintain the low pH level (Chan and Mauro, 2011). There is an association between the lower pH level and the reduced expression of proteins, in particular, those involved in adherence to the skin by *S. aureus* (e.g., protein A, clumping factor B, fibronectin-binding protein A) (Leung, 2013). Therefore, changes in the pH level toward more alkaline are one of the factors, facilitating *S. aureus* colonization and growth in AD patients (O’Regan and Irvine, 2008; Proksch et al., 2008; Clausen et al., 2019). The pH 7.0–8.0 has been shown optimal for *S. aureus* adhesion to human keratinocytes (Mempel et al., 1998).

## No Major *S. Aureus* Clones Could Be Assigned to Isolates From AD Patients

Due to the frequent occurrence of *S. aureus* in patients with AD, as well as the growing number of scientific reports on the mechanisms of immune response induction by specific virulence factors, a natural question arises whether there are selected *S. aureus* clones/types associated with the disease. A “gold standard” to determine the clonality of *S. aureus* strains, especially MRSA, is PFGE (Pulsed Field Gel Electrophoresis) (He et al., 2014). A specific “DNA fingerprint” for an individual clone is assigned to a specific pulsotype (e.g., A, B, C) (Golding et al., 2015). PFGE genotyping of *S. aureus* indicated that among the isolates from AD children, the most common pulsotype was B (48%). In contrast, pulsotype A was the most frequent in the healthy control group (64%) (Lo et al., 2010). However, Lomholt et al. (2005) indicated that 28 various *S. aureus* PFGE pulsotypes could be reported for AD patients.

With the application of MLST (multi-locus sequence typing), that is another useful method of microbial genotyping, Kim et al. (2009) revealed that sequence types ST188, ST1, ST5, and ST513 were the most frequently identified in the studied adolescent or adult patients with AD (19.4, 13.9, 11.1, and 11.1%, respectively). These data demonstrated the absence of prevailed genotype. Additionally, most of the detected lineages (especially ST188 and ST1) were community-acquired strains in contrast to only a single ST5, which in Korea is associated with hospital-acquired strains.

Clausen et al. focused on the distribution of clonal complexes (CCs) determined based on *spa* typing among strains isolated from AD patients. As much as 92% of *S. aureus* isolates demonstrated identical *spa* types in three studied sites (nose, lesional, non-lesional skin). The most frequent *spa* types were t008, t084, t127, and t948 (Clausen et al., 2017). Different results were obtained by Kim et al. (2009), who identified t189 (19.4%) as the most frequent type, followed by t127 (13.9%), t164 (11.1%), and t304 (8.1%). These results confirm the observation on the heterogeneity of *S. aureus* strains isolated from AD patients.

Applying yet another typing method, namely, CC typing, Yeung et al. (2011) proved that in AD patients (adults and children), the most common clonal complex was CC45 (34 of *S. aureus* isolates out of 160), CC5 (23 isolates), CC15 (22 isolates), CC1 (21 isolates), CC30 (11 isolates), and CC398 (8 isolates). In another study by Rojo et al. (2014) two groups of patients were included as follows: AD patients (*n* = 32) and patients who suffered from other atopic diseases (asthma, allergic rhinitis or food allergy, *n* = 31). Among AD patients, the most frequent CC was CC5 (31.2%), CC15 (18.7%), CC30 (18.7%), and CC45 (15.6%), whereas CC30 mostly prevailed in the control group (48.3%) (Rojo et al., 2014). Also, it was demonstrated that 95% of examined samples from AD patients belonged to the same clonal complex in three sampling sites (nose, lesional and non-lesional skin). Interestingly, the authors observed that CC1 was identified more frequently in patients with filaggrin mutations (Clausen et al., 2017). Similarly, Harkins et al. found in the inflamed skin of children with AD that the most prevalent clonal complex was CC1 (20%), whereas CC30 (33%), and CC45 (22%) were predominantly detected in the anterior nares of healthy children (Harkins et al., 2018).

Temporal variation of CC types in *S. aureus* was observed in patients with mild to moderate AD where 52% of patients examined during follow up study were colonized by the same CC type. Interestingly, nearly half of the studied patients (48%) demonstrated different CC types during the follow-up study, which correlated with increased SCORAD (Clausen et al., 2019).

Genetic variations present in *S. aureus* has been shown to influence clinical outcome in some essential diseases (Messina et al., 2016). The connection between clonal complex and infections was documented for CC8 associated with sepsis, CC30 associated with endocarditis or CC398 associated with nasal carriage and bone and joint infection (Nienaber et al., 2011; Spaulding et al., 2012; Valour et al., 2014). The distribution of staphylococcal clonal complexes in AD patients that has been analyzed throughout the recent years points for great heterogeneity, and no specific clone/clones prevailed in this group of patients. Moreover, observations proved that populations of *S. aureus* isolated from AD patients are very clonal, and that characteristic virulence factor variants that have been shown to contribute to AD may occur in different clonal lineages. This further means that this is rather unlikely to characterize specific *S. aureus* lineages associated with AD and disease severity, at least based on traditional typing methods (Kim et al., 2009; Yeung et al., 2011; Rojo et al., 2014; Clausen et al., 2017). The only example of a correlation between *S. aureus* genetic background and AD was the one found by Clausen et al., where CC1 clone was the most commonly detected among AD patients (22% of all colonized patients) and significantly more prevalent in filaggrin mutation carriers (Clausen et al., 2018). Nevertheless, considering the above observation and the dynamic evolution of the *S. aureus* species based on the survival of only those populations that can survive in given conditions (e.g., in a defective atopic skin), the existence of AD-specific genotype(s) cannot be excluded. *S. aureus* genetic variations that might contribute to a particular clinical outcome (like infection of atopic skin) might be present at different levels: clonal, gene, or at the level of gene polymorphisms. Therefore, detailed knowledge about the bacterial genetic variation is needed to understand better the role of *S. aureus* in the pathogenesis of AD.

## A New Concept of Strain-Specific Colonization of *S. Aureus* in AD

Traditional genotyping methods allow the differentiation of staphylococcal isolates from AD patients. These methods, however, have their resolution limitations, which did not allow identification of specific genetic features of AD-derived *S. aureus*. At the same time, there are functional differences manifested by altered immune responses in the skin between AD *S. aureus* strains vs. non-AD *S. aureus* strains. It was observed that only AD-derived *S. aureus* strains altered T cell response via Langerhans cells (Iwamoto et al., 2017), and only AD-derived strains accumulated in lysosomes and induced IL-1α production via Toll-like receptor 9 (Moriwaki et al., 2019). The observed differences have been attributed to surface proteins (Iwamoto et al., 2017; Moriwaki et al., 2019). In line with those reports, *S. aureus* isolated from AD but not from healthy carriers induced strong inflammation in the mouse model of AD (Byrd et al., 2017). Whole-genome sequencing of AD-derived *S. aureus* revealed genes coding for proteins associated with infection, carotenoid production, or β-lactam resistance to be associated with AD colonization (Byrd et al., 2017). Analyzing AD microbiome suggests that AD patients may be preferentially colonized by those *S. aureus* strains that can synthesize tryptophan (Fyhrquist et al., 2019). It was experimentally shown, however, that tryptophan metabolites on atopic skin are significantly reduced, and therefore strains that do not require exogenous tryptophan for growth may be preferred (Yu et al., 2019). The concept of specific *S. aureus* isolates that have a more significant potential to colonize atopic skin or induce an immune effect in AD patients has developed significantly in recent years. Mainly due to the results of studies linking the production of *S. aureus* δ-toxin with allergic skin diseases (Nakamura et al., 2013).

**FIGURE 3:**
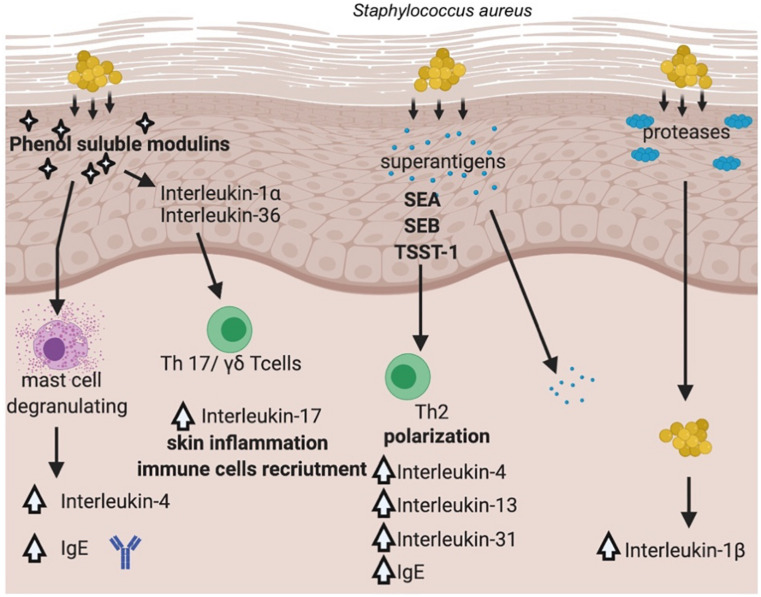
Pathways involved in *S. aureus*’ virulence factors contribution to AD. (Created with BioRender).

*Staphylococcus aureus* once established on the skin, promotes inflammation through multiple pathways. Recent work from several laboratories has advanced our understanding of how the skin colonization of *S. aureus* promotes inflammatory skin diseases. The general overview of the pathways involved in *S. aureus* and its virulence factors contribution to AD is presented in Figure 3.

It has been documented that *S. aureus* is able not only to colonize the surface of the skin, but it also penetrates the dermis, where the bacterium can come into direct contact with immune cells and stimulate the production of proinflammatory cytokines (Nakatsuji et al., 2016). *S. aureus* produces a range of potent virulence factors that appeared to play a crucial role in the inflammation process driven by the bacterium, e.g., PSMs (phenol soluble modulins), proteases (aureolysin, V8 protease, SspA serine protease, ScpA cysteine protease), superantigens (staphylococcal enterotoxin A, B, TSST-1). PSMα has been shown to induce expression of cytokines in keratinocyte cell lines as well as in a mouse model of AD via lysis of keratinocytes, which led to the release of inflammatory cytokines (Syed et al., 2015). Another PSM representative, namely δ-toxin was identified in abundant amounts in culture supernatants of *S. aureus* isolated from the skin of AD patients and was shown to be a potent inducer of mast cells degranulation suggesting for the first time a link between *S. aureus* colonization and allergic skin diseases (Nakamura et al., 2013). In this case, the mechanism of action was different from other PSMs, as δ-toxin (PSMγ) did not cause cell lysis but rather induced signaling pathway leading to increased IgE, IL-4 levels (Figure 3). PSMs are critical for the induction of IL-17 producing cells, namely γδTcells or ILC3 (type 3 lymphoid cells), which are mediators of skin inflammation in response to *S. aureus* (Nakagawa et al., 2017). Depending on the depth bacteria can reach in the skin–epidermis vs. dermis, different host response can be elicited. Epicutaneous exposure of *S. aureus* promotes inflammation via IL-36, produced mostly by keratinocytes, whereas intradermal challenge promotes IL-1β induction of inflammation (Liu et al., 2017). The penetration depth has been shown to critically depend on an important group of virulence factors produced by the bacterium, namely serine proteases (Nakatsuji et al., 2016).

Toll-like receptors (TLRs), recognizing various bacterial antigens (e.g., cell wall components), transduce a signal through MyD88 (Myeloid differentiation primary response gene-88) signaling pathway that leads to activation of NFκB transcription factor and production of proinflammatory cytokines (Kuo et al., 2013). Recently, MyD88-dependent signaling was demonstrated a critical pathway activated in response to staphylococcal virulence factors–PSMα (Liu et al., 2017) and SEB (Faßbender et al., 2017). SEB is one of the best-studied enterotoxins in the context of inflammation in AD patients, next to SEA and TSST-1. However, in recent years, experimental data on other members of this group of virulence factors have emerged, expanding our understanding of the mechanisms linking *S. aureus* and AD (Aziz et al., 2019; Orfali et al., 2019). A transcriptomic approach to study keratinocyte response to SEB or TSST-1 has been shown to up- or downregulate more than 3,000 genes, confirming a previously proposed signaling pathway through CD40 receptor (Schlievert et al., 2020).

These phenomena require even more detailed knowledge, but it turns out that *S. aureus* may play a vital role in the development of AD in a strain-specific manner. The experimental data trying to provide an answer to a question whether *S. aureus* causes AD or its increased survival on AD skin is a consequence of the disease is only starting to emerge. More and more puzzles add up to a complete picture that may soon allow us to understand better the molecular mechanism of a complex relation between *S. aureus* and AD (Geoghegan et al., 2018).

## An Ambiguity of Staphylococcal Enterotoxins (SEs) in Atopic Dermatitis

From the vast repertoire of *S. aureus’* virulence factors, we will focus on a specific group–enterotoxins, which *S. aureus* produces dozens of varieties.

**FIGURE 4:**
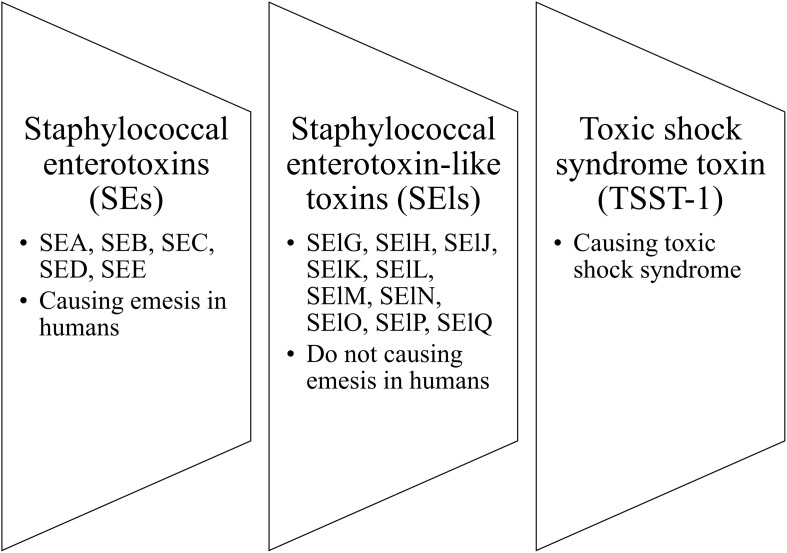
Three clusters of staphylococcal toxins.

The pyrogenic toxin superantigen (PTSA) family is the group of staphylococcal toxins that includes the following clusters: staphylococcal enterotoxins (SEs), staphylococcal enterotoxin-like toxins (SEls), and toxic shock syndrome toxin (TSST-1). The nomenclature distinguished SEs (SEA, SEB, SEC, SED, SEE) from SEls (SElG, SElH, SElJ, SElK, SElL, SElM, SElN, SElO, SElP, and SElQ) is based on their causing (SEs) or not causing (SEls) emesis in humans (Figure 4; Lina et al., 2004). Staphylococcal enterotoxins (SEs) are known as bacterial virulence factors that contribute to the development of many human diseases, including toxic shock syndrome or food poisoning (Harris et al., 1993; Balaban and Rasooly, 2000; Ortega et al., 2010; Pinchuk et al., 2010). Many authors have indicated a role for SEs in the course of AD by acting as factors aggravating and exacerbating the inflammation of AD skin (Bunikowski et al., 2000; Taskapan and Kumar, 2000; Yarwood et al., 2000; Zollner et al., 2000). Moreover, there are indications for the causative role of SEs in the course of AD.

The most common feature of SEs is that they possess superantigenic properties (Spaulding et al., 2013). Superantigens (SAgs) can bind as intact proteins to the T-cell antigen receptor (TCR) and the major histocompatibility complex II (MHC II) outside their binding site (Fink et al., 1986) thus, stimulating massive proliferation of nonspecific T cells and release of proinflammatory cytokines (Harris et al., 1993; Holtfreter et al., 2006).

Because of interest in SEs as an aggravating factor in AD, their distribution in AD-derived *S. aureus* isolates was a matter of deep interest. The main question researchers asked concerned a potential pattern (universal vs. specific) of SEs presence in AD-derived *S. aureus*. It has been known that 54–71.25% of *S. aureus* isolates indicated the presence of SE genes among AD patients (Mempel et al., 2003; Nada et al., 2012). Furthermore, a meta-analysis of 95 studies indicated that the rate of toxins producing *S. aureus* on the lesional skin fluctuated between 31.5 and 80% (Totté et al., 2016), and was higher than in healthy controls. The distribution of SE genes, however, varied from one studied population to another: in Korean population *sea* and *tsst-1* were the most common toxin genes (Kim et al., 2009; Na et al., 2012); in Germany, whereas one study showed that *seb* (38%) and *tsst-1* (23%) toxin genes were the most prevalent, and *sea* was not detected (Zollner et al., 2000), other revealed that *sea* and *sed* were most commonly detected (Breuer et al., 2002). All SEs production patterns from adult AD patients are summarized in Table 2. In the studies of Taiwanese children with AD, the most frequent toxin gene was *seb* (87%), followed by *sec* (9%), *tsst-1* (9%), *sea* (4%), *seg/sei* (4%), and *seh* (4%). The *sed* toxin gene was not identified in atopic children, whereas it was predominant in healthy children. It is worth to mention, however, that only the MRSA population was studied (Lo et al., 2010). In England, the most prevalent toxins were *sec* (25% from the skin; 11% from the nose), *seg* (21% from the skin; 17% from the nose), and *sei* (21% from the skin; 17% from the nose). Most cases represented the same strains in the nose and skin (Arkwright et al., 2001). In Spain, *hla* and *hlg/hlgV* (26, 100%), *hlb* (17, 65.5%), *lukDE* (25, 96.1%), *tsst-1* (13, 50%), *aur* (11, 42.3%), *cna* (10, 38.5%), *eta* (4, 15.4%), and *sec* (2, 7.7%) were the virulence genes detected in cutaneous isolates of children with AD (Gilaberte et al., 2015). The observed variations in SEs distribution patterns in different research and different AD populations indicate geographical dependence. All SEs production patterns from AD children are summarized in Table 1.

Interestingly, in some studies based on populations of both children and adults, the most frequently detected genes were not classical toxins found in 38% of AD-derived strains, but enterotoxin gene cluster (*egc*), which consisted of *seg, sei, sek, sem, sen*, and *seo* (Mempel et al., 2003). Studies on the AD cohort from Singapore indicated that the most prevalent SEs were *seb* (42%), *egc* (32%), and *seh* (29%). Interestingly, Chiu et al. proposed that patients with a moderate type of AD were more likely to be colonized by *S. aureus* possessing staphylococcal enterotoxin B (*seb*) than patients with a severe type of AD (Chiu et al., 2009). Nevertheless, another distribution pattern of toxins was observed in Portuguese AD patients, where 76% of the examined *S. aureus* strains were SE-positive, mainly for *SEls*: *sel-m* and *sel-n* (71.4%), followed by *sel-o* and *seg* (66.7%) and *sea* and *sel-l* were less frequent (29% and 33%, respectively) (Soares et al., 2013). In the Egyptian cohort, the most prevalent gene from the *S. aureus* strains isolated from the lesional skin of AD patients was *seb*, followed by *sec* and *tsst-1*, *sea* and *sed* (Nada et al., 2012). All SEs gene presence patterns from the mixed groups of AD patients are summarized in Table 3.

**TABLE 3:** The distribution of *S. aureus* colonization and toxins production in combined groups of AD patients (both **children and adults**).

References	Examined groups	Sites of isolation	Colonization of *staphylococcus aureus*	Toxins production
Leung et al. (1993) United States	Patients with AD (*n* = 56) Four groups of control patients: - healthy, non-atopic patients (*n* = 15) - patients with psoriasis (*n* = 16) - patients with respiratory allergy: allergic rhinitis and asthma (*n* = 10) - patients with HIE syndrome (*n* = 7)	AD lesional skin	No data	AD patients *sea*: 7 (29%), *seb*: 8 (33%), *sec*: 0 (0%), *sed*: 1 (4%), *tsst-1*: 7 (29%)
Nomura et al. (1999) Japan	Patients with AD (aged 1–22 years *n* = 39)	Exudative dermatitis Dry dermatitis Normal skin	37 of 39 patients (95%) were colonized with *S. aureus* on the skin	21 of 37 *S. aureus* isolates (56.76%) were produced exotoxins *sea*: 2 (5.1%), *seb*: 13 (33.3%), *sec*: 1 (2.6%), *sed*: 1 (2.6%), *tsst-1*: 1 (2.6%), *sea+seb*: 1 (2.6%), *seb+sec*: 1 (2.6%), *seb+tsst-1*: 1 (2.6%)
Matsui et al. (2000) Japan	Patients with AD (aged 7–45, *n* = 26) Healthy group (*n* = 49)	Lesional Non-lesional skin (face, neck or arm)	96.2% of AD patients on the lesional skin 30.8% of AD patients on the non-lesional skin 10.2% of healthy control subjects on the non-lesional skin	Lesional skin in AD patients (40%, 10/25 patients) *sea* (4/10, 40%), *seb* (1/10, 10%), *sec* (5/10, 50%), *sed* (0/10, 0%), *see* (0/10, 0%), *tsst-1* (3/10, 30%) Non-lesional skin in AD patients (37.5%, 3/8 patients) *sea* (0/8, 0%), *seb* (1/8, 12.5%), *sec* (1/8, 12.5%), *sed* (0/8, 0%), *see* (0/8, 0%), *tsst-1* (3/8, 37.5%) Healthy control group (40%, 2/5 patients) *sea* (2/5, 40%), *seb* (0/5, 0%), *sec* (0/5, 0%), *sed* (0/5, 0%), *see* (0/5, 0%), *tsst-1* (1/5, 20%)
Mempel et al. (2003) Germany	Patients from 5 months to 65 years	Eczematous lesions Anterior nares Axillae	80 of 91 patients (87.9%) were colonized with *S. aureus*	120 *S. aureus* strains were tested *sea*: 14 (12%), *seb*: 7 (6%), *sec*: 25 (21%), *sed*: 9 (8%), *see*: 0 (0%), *egc*: 58 (48%), *seh*: 0 (0%), *tsst-1*: 8 (7%)
Yagi et al. (2004) Japan	Patients with AD (*n* = 100) included: - infants (*n* = 55) - children (*n* = 28) - adults (*n* = 17)	No data	No data	81 isolates (81%) produce at least one toxin - nasal cavity: 57(70.4%) - non-lesional area: 33(40.7%) - dry-lesional area: 50(61.7%) - exudative-lesional area: 61(75.3%) *sea*: 22 (27.2%), *seb*: 44 (54.3%), *sec*: 22 (27.2%), *sed*: 4 (4.9%), *tsst-1*: 13 (16.0%)
Tomi et al. (2005) Austria	Patients with AD (3 months-60 years, *n* = 25) Healthy control (*n* = 25)	Lesional skin or volar site of the elbow Nares area	Atopic dermatitis patients 22/25 patients (88%) - skin only: 0 (0%) - nares only: 7 (32%) - skin and nares: 15 (68%) Healthy control 3/25 patients (12%) - skin only: 1 (33%) - nares only: 2 (67%) - skin and nares: 0 (0%)	Atopic dermatitis patients *sec > seb > sea+sed* Healthy control None of the strains were toxigenic
Chiu et al. (2009) Singapore	Children and adults with AD, 2–21 years old (*n* = 34)	Nasal swabs Affected skin	91% were colonized with *S. aureus* (31/34 of isolates) 85% were colonized both in the skin and in the nose (29/34 *S. aureus* strains)	*sea*: 6 (17.6%), *seb*: 13 (38.2%), *sec*: 4 (11.8%), *sed*: 3 (8.8%), *egc*: 10 (29.4%), *seh*: 9 (26.5%), *sek*: 5 (14.7%), *sel*: 2 (5.9%), *tsst-1*: 1 (2.9%)
Na et al. (2012) Korea	Patients with AD (*n* = 39), 1–40 years Healthy control (*n* = 40)	Antecubital area Popliteal fossa Nasal mucosa	Atopic dermatitis patients: - antecubital area: 10(25.6%) - popliteal fossa: 14(35.8%) - nasal mucosa: 18(46.1%) - overall colonization: 25(64.1%) Healthy control: - antecubital area: 1(2.5%) - popliteal fossa: 2(5%) - nasal mucosa: 7(17.5%) - overall colonization: 8(20%)	*sea*: 52.6% *sea+tsst-1*: 42.1% *tsst-1*: 5.3%
Nada et al. (2012) Egypt	AD patients (5–26 years, *n* = 30) Healthy control group (*n* = 30)	From different lesions	AD patients 26 (87%) patients were colonized by *S. aureus*	14 isolates (54%) of 26 *S. aureus* strains produced exotoxins with superantigenic properties *sea*: 1, *seb*: 8, *sec*: 4, *sed*: 1, *tsst-1*: 4
Soares et al. (2013) Portugal	AD patients (3–35 years, *n* = 9) Healthy controls (*n* = 24)	AD patients Samples were collected from the antecubital and popliteal crease Healthy controls Samples were collected from antecubital crease	No data	AD patients (21 *S. aureus* strains were tested) – 16 (76%) were toxigenic *sea*: 6 (29%) *seg*: 14 (66.7%) Other classical toxins (*seb-see*) were not detected.
Rojo et al. (2014) Spain	Patients with AD and colonized by *S. aureus* on lesional skin (*n* = 32) Controls–atopic patients with active allergic disease (asthma, food alergy or rhinitis) and with *S. aureus* isolated from nose (*n* = 31)	Skin area Nasal area Inguinal area Perianal area	All of the patients included in the study were colonized by *S. aureus*	AD patients *sea*: 14 (43.7%), *seb*: 0, *sec*: 7 (21.8%), *see*: 0, *tsst-1*: 4 (12.5%) Atopic controls *sea*: 14 (45.1%), *seb*: 3 (9.6%), *sec*: 2 (6.4%), *see*: 0, *tsst-1*: 15 (48.4%)
Merriman et al. (2016) United States	AD patients (6–37 years, *n* = 103)	Lesional AD skin	No data	*sea*: 7 (6.8%), *seb*: 6 (5.8%), *sec*: 11 (11%), *sed*: 16 (16%), *tsst-1*: 10 (9.7%)

It is not uncommon that AD-derived strains of *S. aureus* can produce more than a single enterotoxin (Schlievert et al., 2008; Na et al., 2012), and at significantly higher amounts (Schlievert et al., 2008). The overall picture is even more complex when SE-specific IgE antibodies are considered. These were detected in AD patients even if the genes coding for particular SEs were absent in *S. aureus*. Furthermore, specific anti-SE IgE was not detected, albeit toxigenic *S. aureus* was isolated from an AD patient (Rojo et al., 2014). Such observation indicated temporal changes in the colonization of AD patients with *S. aureus* (Merriman et al., 2016).

Similarly to the association between the presence of *S. aureus* and AD severity, some authors have also observed that SE-producing *S. aureus* is more associated to AD severity than SE non-producing *S. aureus* (Bunikowski et al., 2000; Zollner et al., 2000; Breuer et al., 2002; Tomi et al., 2005). On the contrary, others found no relationship between superantigens production and the SCORAD index (Mempel et al., 2003; Rojo et al., 2014). These differences may result from various genes repertoire studied by different authors, e.g., Mempel et al. (2003) and Rojo et al. (2014) included enterotoxins rarely studied by others (*see, seh, sej*, and *egc*). Rojo et al. (2014) proved that 65.6% of *S. aureus* strains isolated from AD patients carried genes encoding staphylococcal enterotoxin-like proteins, which function is still poorly understood. Several factors, including estimation of the skin inflammation, the number of patients in the studied groups, or methodological variations in SEs detection (molecular vs. immunological), could be responsible for these divergent observations. In fact one study found that low serum vitamin D levels have been associated with the presence of virulence factors *tsst-1*, *eta*, *can*, *aur*, and *sec* (Gilaberte et al., 2015). Based on the available literature data and the different methodologies used in the studies, it can be concluded that there is a divergent distribution rather than the specific pattern of enterotoxins producing strains among patients with AD.

## Methodology Problems Contributing to the Observed Divergent Distribution of SEs

The presented review attempts to explain possible gaps that may lead to ambiguous results and observations as to whether S. *aureus* isolated from AD patients has any specific features. Among these ambiguities (i) differences in sampling, (ii) isolations site, (iii) characteristics of the population studied, (iv) selection of the control population, and finally (v) the diagnostic method used to detect *S. aureus* and its virulence factors, can be mentioned.

Most researchers used swabs from the different site of AD patients (anterior nares, lesional, and non-lesional skin), while others used contact plate method (Bunikowski et al., 2000; Matsui et al., 2000; Kim et al., 2009). The contact plate method is mainly intended for environmental monitoring of surfaces and is not routinely used in dermatology. The advantage of using a contact plate is a large sampling area compared to the swab method. This allows for reliable quantitative results to be obtained. The second difference is that the contact plate method is swift—sampling and inoculation are performed in one step. However, this method does not allow to sample the anterior nares. On the contrary, the swab method is most commonly used but requires inoculation on agar plates, and sampling covers only a small area of skin. As a result, significant changes in bacterial flora can be overlooked. There is currently no literature evidence comparing the results obtained from these two techniques—swab method and contact plate method in AD patients.

Another problem is the site of bacterial isolation. Most often, samples are taken from AD patients skin, either lesional or non-lesional, or both. On the other hand, anterior nares are not included in many studies, while the nose has been proven to be an important reservoir for *S. aureus* in patients with AD. Also, *S. aureus* can be easily diffused on the skin area due to autotransmission (Breuer et al., 2002). Some studies deserve special attention because they are based on samples of *S. aureus* derived from the skin (lesional and non-lesional) and anterior nares, which allows a better understanding of the distribution of *S. aureus* and its toxins from various sites of isolation (Breuer et al., 2002; Pascolini et al., 2011; Clausen et al., 2019).

The third important issue is the selection of the studied population. For example, some authors based their research on the mix groups of AD patients, that included both children and adults (Matsui et al., 2000; Zollner et al., 2000; Chiu et al., 2009; Nada et al., 2012). It is worth mentioning that presenting results from the mixed group of AD patients may produce different results as if only specific age groups were considered. This is because the course of AD is divided into three phases: infantile, childhood, and adulthood. These phases differ in the location of skin lesions, which may affect the results regarding the distribution of *S. aureus* (Nutten, 2015). Also, aging-related changes in bacterial flora and the distribution of *S. aureus* toxin genes were observed. As an example, regarding the distribution of the *seb* toxin gene, in infants, the s*eb* gene was detected in 46.5% of *S. aureus* strains, in children in 58.3%, and adults in 71.4% strains (Yagi et al., 2004). It should be emphasized here that the number of the studied groups varies considerably from study to study, and often the groups are small, the majority include less than 50 AD patients. This approach may not allow general conclusions to be drawn about the variability of *S. aureus* toxin genes distribution across the entire population.

The problem with the control group is also important and might affect the overall picture of the studied phenomenon. Some studies have omitted the control group (Arkwright et al., 2001; Breuer et al., 2002; Mempel et al., 2003; Kim et al., 2009; Merriman et al., 2016), while the vast majority included healthy, non-atopic, not having accompanying allergic diseases patients, which is the most reasonable approach (Bunikowski et al., 2000; Matsui et al., 2000; Yagi et al., 2004; Pascolini et al., 2011; Nada et al., 2012). However, patients with urticaria, healthy *S. aureus* vagina isolates, or atopic patients with active allergic diseases were also examined as control populations (Schlievert et al., 2008; Park et al., 2013; Rojo et al., 2014). It may be more difficult to observe significant differences or characteristic trends based on the distribution of *S. aureus* toxins in patients with atopic dermatitis and a group of patients with other allergic diseases.

The last problem is the use of various diagnostic methods to detect the studied strain of *S. aureus* and its virulence factors, e.g., staphylococcal enterotoxin genes. The most popular and the most commonly used method for the identification the *S. aureus* toxin genes is PCR (polymerase chain reaction). However, many publications do not contain information on positive and negative controls used in PCR reactions (Yagi et al., 2004; Na et al., 2012). It was uncertain whether the PCR reaction was carried out following approved diagnostic standards. Only a few published papers clearly described the positive controls used in the study (Arkwright et al., 2001; Nada et al., 2012; Merriman et al., 2016). In addition to the molecular method, immunoassay was also used. To detect *S. aureus* enterotoxins, the technique of reversed passive latex agglutination technique (RPLA) was used, which gives semi-quantitative results (Bunikowski et al., 2000; Breuer et al., 2002; Lomholt et al., 2005). As recommended by the Thermo Scientific^TM^, this technique is useful for detecting enterotoxins in a wide variety of foods.

In order to obtain comparable results, the steps should be standardized, from sample preparation to selection of studied groups to a uniform methodology. A universal methodology should be created that can be used to compare results among different laboratories and countries.

## Treatment of Atopic Dermatitis

**FIGURE 5:**
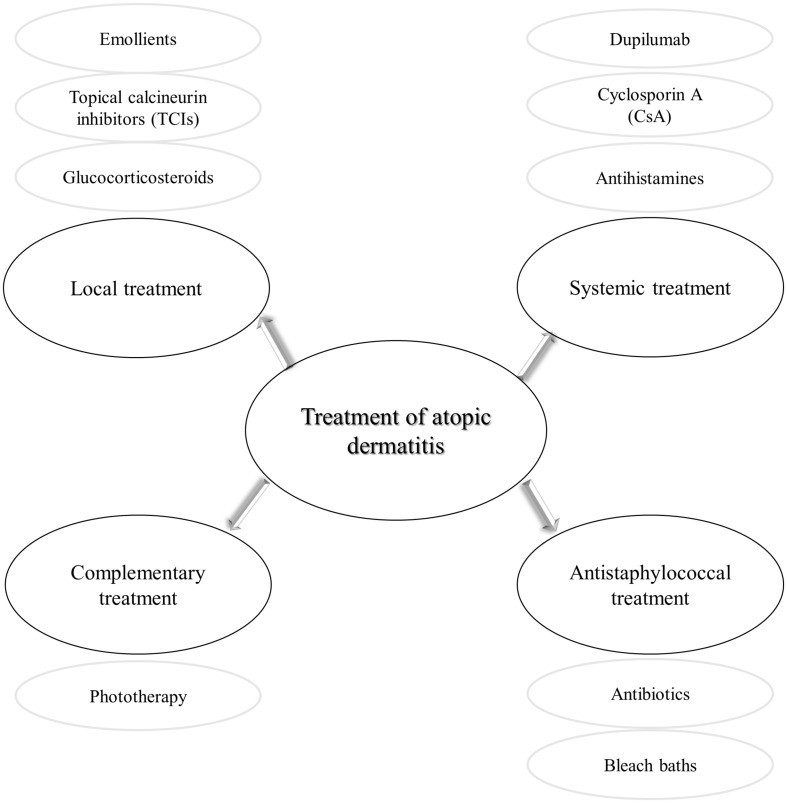
Different types of atopic dermatitis treatment.

The treatment of AD given its multifactorial pathogenesis and chronic characteristics is challenging. It consists of anti-inflammatory and symptomatic therapy, followed by strengthening the damaged natural layers of skin (Figure 5). Local treatment is based on the application of corticosteroids or calcineurin inhibitors, as well as emollients. Topical glucocorticosteroids are the first-line anti-inflammatory treatment in AD (Darsow et al., 2005). These drugs are not free from side effects, e.g., atrophy, purpura, dyspigmentation (Weidinger et al., 2017). As a result of repeated administration of glucocorticosteroids, the response to these compounds is reduced (Brazzini and Pimpinelli, 2002). Anti-inflammatory therapy, free of steroids enables topical calcineurin inhibitors (TCIs), e.g., pimecrolimus (ASM 981/Elidel) and tacrolimus (FK-506/Protopic) (Hanifin et al., 2004; Akdis et al., 2006). Since 2002, an ointment containing tacrolimus has been available in the European Union countries (Rustin, 2007). The central role of TCIs is inhibition of T lymphocytes activation and thus release the inflammatory cytokines (Hanifin et al., 2004). In order to restore the proper barrier function of the damaged skin, it is essential to maintain the disturbed hydration of the epidermis. To this end, moisturizers containing emollients and humectants have been shown to reduce itching, flares and even reduce the necessity of anti-inflammatory drugs (Zuuren et al., 2017). More recently, a targeted topical therapy based on the use of crisaborole 2% ointment has been approved by the FDA to treat children and adults with mild to moderate AD. Crisabolol action is based on inhibiting intracellular enzyme phosphodiesterase 4 to regulate inflammation (Eichenfield et al., 2017; Woo and Kuzel, 2019). As for the topical types of treatment, phototherapy has been considered an option, based on narrow-band UVB or UVA ultraviolet light (Garritsen et al., 2014). This type of treatment is not free from adverse effects, including photodamage or in long-term effects skin carcinogenesis mainly due to the application of UV light (Rodenbeck et al., 2016).

In exceptional cases, cyclosporine A (CsA), methotrexate, azathioprine are used systemically. CsA is a calcineurin inhibitor approved for severe AD cases. Unfortunately, its use is limited (approved only in some EU countries, for patients > 16 y.o.), due to a wide range of side effects, e.g., hypertension, gastrointestinal discomfort, liver dysfunction, and impairment of renal function (with increasing creatinine and urea level in serum) (Hanifin et al., 2004; Akdis et al., 2006; Nowicki et al., 2015). More recently, therapy has been developed that targets the dominant mechanism involved in AD, namely activation of Th2 immune pathways. Dupilumab, a fully human monoclonal antibody targets interleukin 4 receptor alpha (IL-4Rα) that inhibits IL-4 and IL-13 signaling (Simpson et al., 2016; Worm et al., 2020). Another group of modern drugs that seem to be very promising in AD treatment are inhibitors of JAK/STAT signaling pathway, that is responsible for controlling cytokine production and regulation (Schwartz et al., 2017). Some representative of this group of drugs, baricitinib, abrocitinib, and upadacitinib are currently in clinical trials (phase 3 completed) with a positive outcome in studied populations (Ferreira et al., 2020; Simpson et al., 2020a,b).

## Antistaphylococcal Treatment in Atopic Dermatitis Patients

Although antimicrobial treatment is not a key treatment strategy, it is implemented in the course of AD due to a tight correlation of *S. aureus* with exacerbation events. Moreover, the state of dysbiosis, in which lack of beneficial variability in skin microbiome during flares of the disease, is characteristic for AD with huge dominance of *S. aureus*, further justifies the use of antistaphylococcal approaches. Treatment of *S. aureus* colonization/infection in AD is mainly based on antibiotic therapy (topical or systemic). However, overuse of antibiotics can contribute to the development of antibiotic-resistant bacterial strains and the loss of effective antibiotic therapy (Hung et al., 2007). Sule et al. (2007) confirmed this hypothesis showing that use of topical fusidic acid by AD patients in the preceding 6 months contributes to the carriage of fusidic acid-resistant *S. aureus* strains. In Ireland, with wide use of fusidic acid for skin infections and infected AD, resistance to this antibiotic was observed in 24% more AD *S. aureus* strains than in non-atopic control (Harkins et al., 2018). The combination of the topical mupirocin ointment and topical steroids in AD patients revealed sound therapeutic effects manifested by the decrease in EASI and *S. aureus* (from 79.8% before treatment to 40.2% after treatment). In moderate and severe AD, the combination of antibiotic and corticosteroid could be beneficial for patients at early stages of the disease. However, similar clinical effects can be obtained using topical corticosteroid alone (Gong et al., 2006).

An alternative approach in the antistaphylococcal treatment is bleach baths with sodium hypochlorite, which do not always bring the expected effect in reducing *S. aureus* colonization in infants, children and adolescents with AD (Hon et al., 2016; Majewski et al., 2019). The complexity of the disease results in a single treatment option being often insufficient to obtain a good.

## Future Perspectives for the Antistaphylococcal Treatment

Decreased variety of commensal skin microbiota and increased *S. aureus* load are a key pathogenic factor in AD flares. Thus eradication of *S. aureus* and restoring the natural bacterial diversity in the skin is a crucial element in AD management, which has only recently been recognized (Hendricks et al., 2019). Topical antibiotic treatment decreases the number of *S. aureus* but clinically, not all patients benefit from such therapy. Besides spreading resistance to commonly applied antibiotics is a major concern nowadays. There can be two routes to restore the diversity of microbiome on the AD skin: (i) reintroducing natural microbial flora on the skin and/or (ii) decreasing the load of *S. aureus*. The examples of the first approach have already been proposed (Nakatsuji et al., 2017) and verified in some preliminary human trials based on the application of some representatives of coagulase-negative staphylococci (Nakatsuji et al., 2018) or yet another species, namely *Roseomonas mucosa* (Myles et al., 2018). The mechanism behind transplanting beneficial microorganisms directly onto the skin might be based on the production of antistaphylococcal compounds by commensal microorganisms or induction of tolerance to a pathogen. The second approach, namely decreasing the number of *S. aureus*, apart from the application of antibiotics, bleach baths or UV-based phototherapy could be obtained by the use of yet another light-based treatment, namely antimicrobial photodynamic therapy (aPDT).

The aPDT method is based on the use of a nontoxic chemical agent that is excited by visible light in the presence of oxygen. As a result of excitation reactive oxygen species (e.g., O_2_^–^, ⋅OH and singlet oxygen (^1^O_2_) are produced, which damage bacterial cell wall and cellular components (DNA, proteins, lipids), finally resulting in microbial cell death (Cieplik et al., 2018). One of the main advantages of this method is the local action, with no damage to the host tissues (Wainwright et al., 2017). It was documented that aPDT degrades protein toxins by their chemical oxidation (Tubby et al., 2009). In contrast to the UV-based phototherapy, aPDT is characterized by a lack of mutagenicity (Grinholc et al., 2015). Several PS, including rose bengal and methylene blue, applied alone or in combination with antibiotics, have shown significant *in vitro* efficacy in eradicating *S. aureus* growing in planktonic form as well as in biofilm (Pérez-Laguna et al., 2017, 2018). Due to the severe problem with *S. aureus* colonization in AD patients, aPDT could be a useful tool in the antistaphylococcal treatment of chronic or/and recurrent infections (Nakonieczna et al., 2019). It is worth mentioning that the effectiveness of the aPDT method does not depend on antibiotic susceptibility patterns and resistance mechanisms.

PDT is approved in Europe for the treatment of actinic keratosis, squamous cell carcinoma in situ, and some forms of basal cell carcinoma. However, as an “off-label” treatment, PDT has also demonstrated efficacy in inflammatory and infectious dermatoses (Queirós et al., 2020). There is also one case report describing aPDI application for skin treatment in an AD patient. PDT using 20% 5-aminolevulinic acid (ALA) cream was applied as a photosensitizer precursor (2 h incubation). The narrow-band red light was used as a source of light (630 nm, 75 J/cm^2^, 10 min.) to treat a patient suffered from a severe lesion of AD located on the wrists, without signs of *S. aureus* colonization. After three sessions, one every other week, a reduction of the lichenification was observed, and the skin began to heal (Pozzi and Asero, 2010). More recently, daylight PDT with 5-aminolevulinic acid gel (Ameluz) was used to treat chronic hand eczema, half of them for atopic dermatitis (Kremer et al., 2020). After three treatments at 2 week intervals, all the patients showed a significant clinical improvement and also in the quality of life, without significant side effects.

The results mentioned above indicated that PDT could be a promising alternative therapy for the treatment of skin inflammation as well as skin colonization in AD patients, where *S. aureus* is a predominant species.

## Conclusion

The main question of this review was whether certain strains of *S. aureus* are more prone to colonize, infect and deteriorate atopic skin. Partially, the answer to this question is yes. MRSA is more often isolated from atopic skin than healthy. However, no specific clone can be assigned to isolates from AD patients, as assessed by classical genotyping methods such as PFGE or MLST. Adaptation of *S. aureus* to live on atopic skin can rather be detected at a strain-specific level and identified by the whole genome sequencing. Characterization of *S. aureus* from AD patients at a strain-specific level is only just beginning to be more available. The exact mechanisms responsible for the interaction of *S. aureus* with the cutaneous immune system has not yet been clarified. However, the latest research results open further fields for exploration and emergence of new strategies of treatment.

## Author Contributions

PO performed literature search and wrote the first draft of the manuscript. YG and WB-R edited, discussed, and organized the text. JN wrote paragraphs concerning photodynamic treatment, concept of strain-specific colonization in AD, conclusions, and edited the final version of the manuscript. All authors contributed to the article and approved the submitted version.

## Conflict of Interest

The authors declare that the research was conducted in the absence of any commercial or financial relationships that could be construed as a potential conflict of interest.

## References

[B1] Abad E. D. de Ferreira D. C. Cavalcante F. S. Saintive S. Goudouris E. Prado E. A. (2019). High incidence of acquiring methicillin-resistant *Staphylococcus aureus* in Brazilian children with atopic dermatitis and associated risk factors. *J. Microbiol. Immunol. Infect.* 53 724–730. 10.1016/j.jmii.2018.12.014 30956127

[B2] Akdis C. A. Akdis M. Bieber T. Bindslev-Jensen C. Boguniewicz M. Eigenmann P. (2006). Diagnosis and treatment of atopic dermatitis in children and adults: European Academy of Allergology and Clinical Immunology/American Academy of Allergy, Asthma and Immunology/PRACTALL consensus report. *Allergy Eur. J. Allergy Clin. Immunol.* 118 152–169. 10.1111/j.1398-9995.2006.01153.x 16815151

[B3] Alsterholm M. Strömbeck L. Ljung A. Karami N. Widjestam J. Gillstedt M. (2017). Variation in Staphylococcus aureus colonization in relation to disease severity in adults with atopic dermatitis during a five-month follow-up. *Acta Derm. Venereol.* 97 802–807. 10.2340/00015555-2667 28374043

[B4] Arikawa J. Ishibashi M. Kawashima M. Takagi Y. Ichikawa Y. Imokawa G. (2002). Decreased levels of sphingosine, a natural antimicrobial agent, may be associated with vulnerability of the stratum corneum from patients with atopic dermatitis to colonization by *Staphylococcus aureus*. *J. Invest. Dermatol.* 119 433–439. 10.1046/j.1523-1747.2002.01846.x 12190867

[B5] Arkwright P. D. Cookson B. D. Haeney M. R. Sanyal D. Potter M. R. David T. J. (2001). Children with atopic dermatitis who carry toxin-positive *Staphylococcus aureus* strains have an expansion of blood CD5- B lymphocytes without an increase in disease severity. *Clin. Exp. Immunol.* 125 184–189. 10.1046/j.1365-2249.2001.01620.x 11529907 PMC1906122

[B6] Aziz F. Hisatsune J. Yu L. Kajimura J. Sato’o Y. Ono H. K. (2019). *Staphylococcus aureus* isolated from skin from atopic-dermatitis patients produces staphylococcal enterotoxin Y, which predominantly induces T-Cell receptor Vα-specific expansion of T cells. *Infect. Immun.* 88:e00360-19. 10.1128/IAI.00360-19 31740530 PMC6977126

[B7] Balaban N. Rasooly A. (2000). Staphylococcal enterotoxins. *Int. J. Food Microbiol.* 61 1–10. 11028954 10.1016/s0168-1605(00)00377-9

[B8] Berth-Jones J. Berth-Jones J. (1996). Six area, six sign atopic dermatitis (SASSAD) severity score: a simple system for monitoring disease activity in atopic dermatitis. *Br. J. Dermatol.* 135(Suppl. 48) 25–30. 10.1111/j.1365-2133.1996.tb00706.x 8881901

[B9] Blome C. Radtke M. A. Eissing L. Augustin M. (2016). Quality of life in patients with atopic dermatitis: disease burden, measurement, and treatment benefit. *Am. J. Clin. Dermatol.* 17 163–169. 10.1007/s40257-015-0171-3 26818063

[B10] Bonamonte D. Filoni A. Vestita M. Romita P. Foti C. Angelini G. (2019). The role of the environmental risk factors in the pathogenesis and clinical outcome of atopic dermatitis. *Biomed Res. Int.* 2019:2450605. 10.1155/2019/2450605 31119157 PMC6500695

[B11] Brazzini B. Pimpinelli N. (2002). New and established topical corticosteroids in dermatology: clinical pharmacology and therapeutic use. *Am. J. Clin. Dermatol.* 3 47–58. 10.2165/00128071-200203010-00005 11817968

[B12] Breuer K. HAussler S. Kapp A. Werfel T. (2002). *Staphylococcus aureus*: colonizing features and influence of an antibacterial treatment in adults with atopic dermatitis. *Br. J. Dermatol.* 147 55–61. 10.1046/j.1365-2133.2002.04872.x 12100185

[B13] Brown S. J. McLean W. H. I. (2012). One remarkable molecule: filaggrin. *J. Invest. Dermatol.* 132(3 Pt 2), 751–762. 10.1038/jid.2011.393 22158554 PMC3378480

[B14] Bunikowski R. Mielke M. E. Skarabis H. Worm M. Anagnostopoulos I. Kolde G. (2000). Evidence for a disease-promoting effect of *Staphylococcus aureus*-derived exotoxins in atopic dermatitis. *J. Allergy Clin. Immunol.* 105 814–819. 10.1067/mai.2000.105528 10756234

[B15] Butaye P. Argudín M. A. Smith T. C. (2016). Livestock-associated MRSA and its current evolution. *Curr. Clin. Microbiol. Rep.* 3 19–31. 10.1007/s40588-016-0031-9

[B16] Byrd A. L. Deming C. Cassidy S. K. B. Harrison O. J. Ng W.-I. Conlan S. (2017). *Staphylococcus aureus* and *Staphylococcus* epidermidis strain diversity underlying pediatric atopic dermatitis. *Sci. Transl. Med.* 9:eaal4651. 10.1126/scitranslmed.aal4651 28679656 PMC5706545

[B17] Candi E. Schmidt R. Melino G. (2005). The cornified envelope: a model of cell death in the skin. *Nat. Rev. Mol. Cell Biol.* 6 328–340. 10.1038/nrm1619 15803139

[B18] Carroll C. L. Balkrishnan R. Feldman S. R. Fleischer A. B. Manuel J. C. (2005). The burden of atopic dermatitis: impact on the patient, family, and society. *Pediatr. Dermatol.* 22 192–199. 10.1111/j.1525-1470.2005.22303.x 15916563

[B19] Chan A. Mauro T. (2011). Acidification in the epidermis and the role of secretory phospholipases. *Dermatoendocrinology* 3 84–90. 10.4161/derm.3.2.15140 21695017 PMC3117007

[B20] Chiesa Fuxench Z. C. Block J. K. Boguniewicz M. Boyle J. Fonacier L. Gelfand J. M. (2019). Atopic dermatitis in America study: a cross-sectional study examining the prevalence and disease burden of atopic dermatitis in the US adult population. *J. Invest. Dermatol.* 139 583–590. 10.1016/j.jid.2018.08.028 30389491

[B21] Chiu L. S. Ho M. S. L. Hsu L. Y. Tang M. B. Y. (2009). Prevalence and molecular characteristics of *Staphylococcus aureus* isolates colonizing patients with atopic dermatitis and their close contacts in Singapore. *Br. J. Dermatol.* 160 965–971. 10.1111/j.1365-2133.2009.09038.x 19222456

[B22] Chung H. J. Jeon H. S. Sung H. Kim M. N. Hong S. J. (2008). Epidemiological characteristics of methicillin-resistant *Staphylococcus aureus* isolates from children with eczematous atopic dermatitis lesions. *J. Clin. Microbiol.* 46 991–995. 10.1128/JCM.00698-07 18174298 PMC2268387

[B23] Cieplik F. Deng D. Crielaard W. Buchalla W. Hellwig E. Al-Ahmad A. (2018). Antimicrobial photodynamic therapy–what we know and what we don’t. *Crit. Rev. Microbiol.* 44 571–589. 10.1080/1040841X.2018.1467876 29749263

[B24] Clausen M. L. Agner T. Lilje B. Edslev S. M. Johannesen T. B. Andersen P. S. (2018). Association of disease severity with skin microbiome and filaggrin gene mutations in adult atopic dermatitis. *JAMA Dermatol.* 154 293–300. 10.1001/jamadermatol.2017.5440 29344612 PMC5885821

[B25] Clausen M. L. Edslev S. M. Andersen P. S. Clemmensen K. Krogfelt K. A. Agner T. (2017). *Staphylococcus aureus* colonization in atopic eczema and its association with filaggrin gene mutations. *Br. J. Dermatol.* 177 1394–1400. 10.1111/bjd.15470 28317091

[B26] Clausen M. L. Edslev S. M. Nørreslet L. B. Sørensen J. A. Andersen P. S. Agner T. (2019). Temporal variation of *Staphylococcus aureus* clonal complexes in atopic dermatitis: a follow-up study. *Br. J. Dermatol.* 180 181–186. 10.1111/bjd.17033 30070683

[B27] Coderch L. López O. De La Maza A. Parra J. L. (2003). Ceramides and skin function. *Am. J. Clin. Dermatol.* 4 107–129. 10.2165/00128071-200304020-00004 12553851

[B28] Darsow U. Lübbe J. Taïeb A. Seidenari S. Wollenberg A. Calza A. M. (2005). Position paper on diagnosis and treatment of atopic dermatitis. *J. Eur. Acad. Dermatol. Venereol.* 19 286–295. 10.1111/j.1468-3083.2005.01249.x 15857453

[B29] Di Nardo A. Wertz P. Giannetti A. Seidenari S. (1998). Ceramide and cholesterol composition of the skin of patients with atopic dermatitis. *Acta Derm. Venereol.* 78 27–30. 10.1080/00015559850135788 9498022

[B30] Eichenfield L. F. Call R. S. Forsha D. W. Fowler J. Hebert A. A. Spellman M. (2017). Long-term safety of crisaborole ointment 2% in children and adults with mild to moderate atopic dermatitis. *J. Am. Acad. Dermatol.* 77 641.e5–649.e5. 10.1016/j.jaad.2017.06.010 28823881

[B31] Faßbender S. Opitz F. V. Johnen S. Förster I. Weighardt H. (2017). MyD88 contributes to staphylococcal enterotoxin B-triggered atopic dermatitis-like skin inflammation in Mice. *J. Invest. Dermatol.* 137 1802–1804. 10.1016/j.jid.2017.04.015 28457911

[B32] Ferreira S. Guttman-Yassky E. Torres T. (2020). Selective JAK1 inhibitors for the treatment of atopic dermatitis: focus on upadacitinib and abrocitinib. *Am. J. Clin. Dermatol.* 21 783–798. 10.1007/s40257-020-00548-6 32776305

[B33] Fink P. J. Matis L. A. McElligott D. L. Bookman M. Hedrick S. M. (1986). Correlations between T-cell specificity and the structure of the antigen receptor. *Nature* 321 219–226. 10.1038/321219a0 3012351

[B34] Fyhrquist N. Muirhead G. Prast-Nielsen S. Jeanmougin M. Olah P. Skoog T. (2019). Microbe-host interplay in atopic dermatitis and psoriasis. *Nat. Commun.* 10:4703. 10.1038/s41467-019-12253-y 31619666 PMC6795799

[B35] Garritsen F. M. Brouwer M. W. D. Limpens J. Spuls P. I. (2014). Photo(chemo)therapy in the management of atopic dermatitis: an updated systematic review with implications for practice and research. *Br. J. Dermatol.* 170 501–513. 10.1111/bjd.12645 24116934

[B36] Geoghegan J. A. Irvine A. D. Foster T. J. (2018). *Staphylococcus aureus* and atopic dermatitis: a complex and evolving relationship. *Trends Microbiol.* 26 484–497. 10.1016/j.tim.2017.11.008 29233606

[B37] Gilaberte Y. Sanmartín R. Aspiroz C. Hernandez-Martin A. Benito D. Sanz-Puertolas P. (2015). Correlation between serum 25-hydroxyvitamin D and virulence genes of staphylococcus aureus isolates colonizing children with atopic dermatitis. *Pediatr. Dermatol.* 32 506–513. 10.1111/pde.12436 25491017

[B38] Golding G. R. Campbell J. Spreitzer D. Chui L. (2015). Pulsed-field gel electrophoresis of *Staphylococcus aureus*. *Methods Mol. Biol.* 1301 85–93. 10.1007/978-1-4939-2599-5_8 25862050

[B39] Gong J. Q. Lin L. Lin T. Hao F. Zeng F. Q. Bi Z. G. (2006). Skin colonization by *Staphylococcus aureus* in patients with eczema and atopic dermatitis and relevant combined topical therapy: a double-blind multicentre randomized controlled trial. *Br. J. Dermatol.* 155 680–687. 10.1111/j.1365-2133.2006.07410.x 16965415

[B40] Grinholc M. Rodziewicz A. Forys K. Rapacka-Zdonczyk A. Kawiak A. Domachowska A. (2015). Fine-tuning recA expression in Staphylococcus aureus for antimicrobial photoinactivation: importance of photo-induced DNA damage in the photoinactivation mechanism. *Appl. Microbiol. Biotechnol.* 99 9161–9176. 10.1007/s00253-015-6863-z 26252968 PMC4619464

[B41] Gruber R. Elias P. M. Crumrine D. Lin T.-K. Brandner J. M. Hachem J.-P. (2011). Filaggrin genotype in ichthyosis vulgaris predicts abnormalities in epidermal structure and function. *Am. J. Pathol.* 178 2252–2263. 10.1016/j.ajpath.2011.01.053 21514438 PMC3081164

[B42] Hanifin J. M. Cooper K. D. Ho V. C. Kang S. Krafchik B. R. Margolis D. J. (2004). Guidelines of care for atopic dermatitis. *J. Am. Acad. Dermatol.* 50 391–404. 10.1016/j.jaad.2003.08.003 14988682

[B43] Hanifin J. M. Rajka G. (1980). Diagnostic features of atopic dermatitis. *Acta Derm. Venereol. Suppl*. 92 44–47.

[B44] Hanifin J. M. Thurston M. Omoto M. Cherill R. Tofte S. J. Graeber M. (2001). The eczema area and severity index (EASI): assessment of reliability in atopic dermatitis. *Exp. Dermatol.* 10 11–18. 10.1034/j.1600-0625.2001.100102.x 11168575

[B45] Harkins C. P. Pettigrew K. A. Oravcová K. Gardner J. Hearn R. M. R. Rice D. (2018). The microevolution and epidemiology of *Staphylococcus aureus* colonization during atopic eczema disease flare. *J. Invest. Dermatol.* 138 336–343. 10.1016/j.jid.2017.09.023 28951239 PMC5780352

[B46] Harris T. O. Grossman D. Kappler J. W. Marrack P. Rich R. R. Betley M. J. (1993). Lack of complete correlation between emetic and T-cell-stimulatory activities of staphylococcal enterotoxins. *Infect. Immun.* 61 3175–3183. 10.1128/IAI.61.8.3175-3183.1993 8335347 PMC280985

[B47] Hauser C. Wuethrich B. Matter L. Wilhelm J. A. Sonnabend W. Schopfer K. (1985). *Staphylococcus aureus* skin colonization in atopic dermatitis patients. *Dermatologica* 170 35–39. 10.1159/000249493 3972149

[B48] He Y. Xie Y. Reed S. (2014). Pulsed-field gel electrophoresis typing of *Staphylococcus aureus* isolates. *Methods Mol. Biol.* 1085 103–111. 10.1007/978-1-62703-664-1_6 31523766

[B49] Hendricks A. J. Mills B. W. Shi V. Y. (2019). Skin bacterial transplant in atopic dermatitis: knowns, unknowns and emerging trends. *J. Dermatol. Sci.* 95 56–61. 10.1016/j.jdermsci.2019.07.001 31395434

[B50] Holtfreter S. Roschack K. Eichler P. Eske K. Holtfreter B. Kohler C. (2006). *Staphylococcus aureus* carriers neutralize superantigens by antibodies specific for their colonizing strain: a potential explanation for their improved prognosis in severe sepsis. *J. Infect. Dis.* 193 1275–1278. 10.1086/503048 16586365

[B51] Hon K. L. Tsang Y. C. K. Lee V. W. Y. Pong N. H. Ha G. Lee S. T. (2016). Efficacy of sodium hypochlorite (bleach) baths to reduce *Staphylococcus aureus* colonization in childhood onset moderate-to-severe eczema: a randomized, placebo-controlled cross-over trial. *J. Dermatolog. Treat.* 27 156–162. 10.3109/09546634.2015.1067669 26270469

[B52] Housman T. S. Patel M. J. Camacho F. Feldman S. R. Fleischer A. B. Balkrishnan R. (2002). Use of the self-administered eczema area and severity index by parent caregivers: results of a validation study. *Br. J. Dermatol.* 147 1192–1198. 10.1046/j.1365-2133.2002.05031.x 12452870

[B53] Hung S.-H. Lin Y.-T. Chu C.-Y. Lee C.-C. Liang T.-C. Yang Y.-H. (2007). *Staphylococcus* colonization in atopic dermatitis treated with fluticasone or tacrolimus with or without antibiotics. *Ann. Allergy. Asthma Immunol.* 98 51–56. 10.1016/S1081-1206(10)60859-9 17225720

[B54] Iwamoto K. Moriwaki M. Niitsu Y. Saino M. Takahagi S. Hisatsune J. (2017). Staphylococcus aureus from atopic dermatitis skin alters cytokine production triggered by monocyte-derived Langerhans cell. *J. Dermatol. Sci.* 88 271–279. 10.1016/j.jdermsci.2017.08.001 28822698

[B55] Kanda N. Watanabe S. (2012). Increased serum human β-defensin-2 levels in atopic dermatitis: relationship to IL-22 and oncostatin M. *Immunobiology* 217 436–445. 10.1016/j.imbio.2011.10.010 22137028

[B56] Kim D.-W. Park J.-Y. Park K.-D. Kim T.-H. Lee W.-J. Lee S.-J. (2009). Are there predominant strains and toxins of Staphylococcus aureus in atopic dermatitis patients? Genotypic characterization and toxin determination of S. aureus isolated in adolescent and adult patients with atopic dermatitis. *J. Dermatol.* 36 75–81. 10.1111/j.1346-8138.2009.00592.x 19284449

[B57] King M. D. Humphrey B. J. Wang Y. F. Kourbatova E. V. Ray S. M. Blumberg H. M. (2006). Emergence of community-acquired methicillin-resistant *Staphylococcus aureus* USA 300 clone as the predominant cause of skin and soft-tissue infections. *Ann. Intern. Med.* 144 309–317. 10.7326/0003-4819-144-5-200603070-00005 16520471

[B58] Kremer N. Sherman S. Lapidoth M. Enk C. D. Leshem Y. A. Mimouni T. (2020). Self-administered daylight-activated photodynamic therapy for the treatment of hand eczema: a prospective proof-of-concept study. *Dermatol. Ther.* e14329. 10.1111/dth.14329 32975350

[B59] Kunz B. Oranje A. P. Labréze L. Stabler J. F. Ring J. Taïeb A. (1997). Clinical validation and guidelines for the scorad index: consensus report of the european task force on atopic dermatitis. *Dermatology* 195 10–19. 10.1159/000245677 9267730

[B60] Kuo I. H. Yoshida T. De Benedetto A. Beck L. A. (2013). The cutaneous innate immune response in patients with atopic dermatitis. *J. Allergy Clin. Immunol.* 131 266–278. 10.1016/j.jaci.2012.12.1563 23374259

[B61] Leung D. Y. Harbeck R. Bina P. Reiser R. F. Yang E. Norris D. A. (1993). Presence of IgE antibodies to staphylococcal exotoxins on the skin of patients with atopic dermatitis. Evidence for a new group of allergens. *J. Clin. Invest.* 92 1374–1380. 10.1172/JCI116711 7690780 PMC288279

[B62] Leung D. Y. M. (2013). New Insights into atopic dermatitis: role of skin barrier and immune dysregulation. *Allergol. Int.* 62 151–161. 10.2332/allergolint.13-RAI-0564 23712284 PMC8609663

[B63] Leyden J. J. Marples R. R. (1973). Ecologic principles and antibiotic therapy in chronic dermatoses. *Arch. Dermatol.* 107 208–211. 10.1001/archderm.1973.01620170020006 4265456

[B64] Lina G. Bohach G. A. Nair S. P. Hiramatsu K. Jouvin-Marche E. Mariuzza R. (2004). Standard nomenclature for the superantigens expressed by *Staphylococcus*. *J. Infect. Dis.* 189 2334–2336. 10.1086/420852 15181583

[B65] Liu H. Archer N. K. Dillen C. A. Wang Y. Ashbaugh A. G. Ortines R. V. (2017). *Staphylococcus aureus* epicutaneous exposure drives skin inflammation via IL-36-Mediated T cell responses. *Cell Host Microbe* 22 653.e5–666.e5. 10.1016/j.chom.2017.10.006 29120743 PMC5774218

[B66] Lo W. T. Wang S. R. Tseng M. H. Huang C. F. Chen S. J. Wang C. C. (2010). Comparative molecular analysis of meticillin-resistant *Staphylococcus aureus* isolates from children with atopic dermatitis and healthy subjects in Taiwan. *Br. J. Dermatol.* 162 1110–1116. 10.1111/j.1365-2133.2010.09679.x 20132206

[B67] Lomholt H. Andersen K. E. Kilian M. (2005). *Staphylococcus aureus* clonal dynamics and virulence factors in children with atopic dermatitis. *J. Invest. Dermatol.* 125 977–982. 10.1111/j.0022-202X.2005.23916.x 16297199

[B68] Majewski S. Bhattacharya T. Asztalos M. Bohaty B. Durham K. C. West D. P. (2019). Sodium hypochlorite body wash in the management of *Staphylococcus aureus*–colonized moderate-to-severe atopic dermatitis in infants, children, and adolescents. *Pediatr. Dermatol.* 36 442–447. 10.1111/pde.13842 30983053 PMC6767696

[B69] Matsui K. Nishikawa A. Suto H. Tsuboi R. Ogawa H. (2000). Comparative study of *Staphylococcus aureus* isolated from lesional and non-lesional skin of atopic dermatitis patients. *Microbiol. Immunol.* 44 945–947. 10.1111/j.1348-0421.2000.tb02587.x 11145276

[B70] Mempel M. Lina G. Hojka M. Schnopp C. Seidl H.-P. Schäfer T. (2003). High prevalence of superantigens associated with the egc locus in *Staphylococcus aureus* isolates from patients with atopic eczema. *Eur. J. Clin. Microbiol. Infect. Dis.* 22 306–309. 10.1007/s10096-003-0928-0 12743832

[B71] Mempel M. Schmidt T. Weidinger S. Schnopp C. Ring J. Abeck D. (1998). Role of *Staphylococcus aureus* surface-associated proteins in the attachment to cultured HaCaT keratinocytes in a new adhesion assay. *J. Invest. Dermatol.* 111 452–456. 10.1046/j.1523-1747.1998.00293.x 9740240

[B72] Merriman J. A. Mueller E. A. Cahill M. P. Beck L. A. Paller A. S. Hanifin J. M. (2016). Temporal and racial differences associated with atopic dermatitis *Staphylococcus aureus* and encoded virulence factors. *mSphere* 1:e00295-16. 10.1128/mSphere.00295-16 27981233 PMC5143412

[B73] Messina J. A. Thaden J. T. Sharma-Kuinkel B. K. Fowler V. G. (2016). Impact of bacterial and human genetic variation on *Staphylococcus aureus* infections. *PLoS Pathog.* 12:e1005330. 10.1371/journal.ppat.1005330 26766507 PMC4713168

[B74] Miajlovic H. Fallon P. G. Irvine A. D. Foster T. J. (2010). Effect of filaggrin breakdown products on growth of and protein expression by *Staphylococcus aureus*. *J. Allergy Clin. Immunol.* 126 1184.e3–1190.e3. 10.1016/j.jaci.2010.09.015 21036388 PMC3627960

[B75] Moriwaki M. Iwamoto K. Niitsu Y. Matsushima A. Yanase Y. Hisatsune J. (2019). *Staphylococcus aureus* from atopic dermatitis skin accumulates in the lysosomes of keratinocytes with induction of IL-1α secretion via TLR9. *Allergy* 74 560–571. 10.1111/all.13622 30269350

[B76] Murata Y. Ogata J. Higaki Y. Kawashima M. Yada Y. Higuchi K. (1996). Abnormal expression of sphingomyelin acylase in atopic dermatitis: an etiologic factor for ceramide deficiency? *J. Invest. Dermatol.* 106 1242–1249. 10.1111/1523-1747.ep12348937 8752664

[B77] Myles I. A. Earland N. J. Anderson E. D. Moore I. N. Kieh M. D. Williams K. W. (2018). First-in-human topical microbiome transplantation with *Roseomonas mucosa* for atopic dermatitis. *JCI Insight.* 3:e120608. 10.1172/jci.insight.120608 29720571 PMC6012572

[B78] Na S.-Y. Roh J.-Y. Kim J.-M. Tamang M. D. Lee J.-R. (2012). Analysis of colonization and genotyping of the exotoxins of *Staphylococcus aureus* in patients with atopic dermatitis. *Ann. Dermatol.* 24:413. 10.5021/ad.2012.24.4.413 23197906 PMC3505771

[B79] Nada H. A. Gomaa N. I. M. Elakhras A. Wasfy R. Baker R. A. (2012). Skin colonization by superantigen-producing *Staphylococcus aureus* in Egyptian patients with atopic dermatitis and its relation to disease severity and serum interleukin-4 level. *Int. J. Infect. Dis.* 16 e29–e33. 10.1016/j.ijid.2011.09.014 22040925

[B80] Nakagawa S. Matsumoto M. Katayama Y. Oguma R. Wakabayashi S. Nygaard T. (2017). *Staphylococcus aureus* virulent PSMα peptides induce keratinocyte alarmin release to orchestrate IL-17-dependent skin inflammation. *Cell Host Microbe* 22 667.e5–677.e5. 10.1016/j.chom.2017.10.008 29120744 PMC5728420

[B81] Nakamura Y. Oscherwitz J. Cease K. B. Chan S. M. Muñoz-Planillo R. Hasegawa M. (2013). Staphylococcus δ-toxin induces allergic skin disease by activating mast cells. *Nature* 503 397–401. 10.1038/nature12655 24172897 PMC4090780

[B82] Nakatsuji T. Chen T. H. Narala S. Chun K. A. Two A. M. Yun T. (2017). Antimicrobials from human skin commensal bacteria protect against *Staphylococcus aureus* and are deficient in atopic dermatitis. *Sci. Transl. Med.* 9:eaah4680. 10.1126/scitranslmed.aah4680 28228596 PMC5600545

[B83] Nakatsuji T. Chen T. H. Two A. M. Chun K. A. Narala S. Geha R. S. (2016). *Staphylococcus aureus* exploits epidermal barrier defects in atopic dermatitis to trigger cytokine expression. *J. Invest. Dermatol.* 136 2192–2200. 10.1016/j.jid.2016.05.127 27381887 PMC5103312

[B84] Nakatsuji T. Yun T. Butcher A. Hayashi A. Chun K. Shafiq F. (2018). 426 Clinical improvement in atopic dermatitis following autologous application of microbiome therapy targeting *Staphylococcus aureus*. *J. Invest. Dermatol.* 138:S72 10.1016/j.jid.2018.03.433

[B85] Nakonieczna J. Wozniak A. Pieranski M. Rapacka-Zdonczyk A. Ogonowska P. Grinholc M. (2019). Photoinactivation of ESKAPE pathogens: overview of novel therapeutic strategy. *Future Med. Chem.* 11 443–461. 10.4155/fmc-2018-0329 30901231

[B86] Nienaber J. J. C. Sharma Kuinkel B. K. Clarke-Pearson M. Lamlertthon S. Park L. Rude T. H. (2011). Methicillin-susceptible *Staphylococcus aureus* endocarditis isolates are associated with clonal complex 30 genotype and a distinct repertoire of enterotoxins and adhesins. *J. Infect. Dis.* 204 704–713. 10.1093/infdis/jir389 21844296 PMC3156104

[B87] Niyonsaba F. Kiatsurayanon C. Chieosilapatham P. Ogawa H. (2017). Friends or foes? Host defense (antimicrobial) peptides and proteins in human skin diseases. *Exp. Dermatol.* 26 989–998. 10.1111/exd.13314 28191680

[B88] Nomura I. Tanaka K. Tomita H. Katsunuma T. Ohya Y. Ikeda N. (1999). Evaluation of the staphylococcal exotoxins and their specific IgE in childhood atopic dermatitis. *J. Allergy Clin. Immunol.* 104 441–446. 10.1016/S0091-6749(99)70390-8 10452768

[B89] Nowicki R. Trzeciak M. Wilkowska A. Sokołowska-WojWojdyło M. Ługowska-Umer H. Barańska-Rybak W. (2015). Atopic dermatitis: current treatment guidelines. Statement of the experts of the dermatological section, polish society of allergology, and the allergology section, polish society of dermatology. *Post. Dermatol. Alergol.* 32 239–249. 10.5114/pdia.2015.53319 26366146 PMC4565838

[B90] Nutten S. (2015). Atopic dermatitis: global epidemiology and risk factors. *Ann. Nutr. Metab.* 66 8–16. 10.1159/000370220 25925336

[B91] Ohnishi Y. Okino N. Ito M. Imayama S. (1999). Ceramidase activity in bacterial skin flora as a possible cause of ceramide deficiency in atopic dermatitis. *Clin. Diagn. Lab. Immunol.* 6 101–104. 9874672 10.1128/cdli.6.1.101-104.1999 PMC95668

[B92] Ong P. Y. Ohtake T. Brandt C. Strickland I. Boguniewicz M. Ganz T. (2002). Endogenous antimicrobial peptides and skin infections in atopic dermatitis. *N. Engl. J. Med.* 347 1151–1160. 10.1056/nejmoa021481 12374875

[B93] O’Regan G. M. Irvine A. D. (2008). The role of filaggrin loss-of-function mutations in atopic dermatitis. *Curr. Opin. Allergy Clin. Immunol.* 8 406–410. 10.1097/ACI.0b013e32830e6fb2 18769192

[B94] O’Regan G. M. Sandilands A. McLean W. H. I. Irvine A. D. (2008). Filaggrin in atopic dermatitis. *J. Allergy Clin. Immunol.* 122 689–693. 10.1016/j.jaci.2008.08.002 18774165

[B95] Orfali R. L. Yoshikawa F. S. Y. da Oliveira L. M. S. Pereira N. Z. de Lima J. F. Ramos Y. ÁL. (2019). Staphylococcal enterotoxins modulate the effector CD4+ T cell response by reshaping the gene expression profile in adults with atopic dermatitis. *Sci. Rep.* 9:13082. 10.1038/s41598-019-49421-5 31511620 PMC6739319

[B96] Ortega E. Abriouel H. Lucas R. Gálvez A. (2010). Multiple roles of *Staphylococcus aureus* enterotoxins: pathogenicity, superantigenic activity, and correlation to antibiotic resistance. *Toxins* 2 2117–2131. 10.3390/toxins2082117 22069676 PMC3153285

[B97] Park H. Y. Kim C. R. Huh I. S. Jung M. Y. Seo E. Y. Park J. H. (2013). *Staphylococcus aureus* colonization in acute and chronic skin lesions of patients with atopic dermatitis. *Ann. Dermatol.* 25 410–416. 10.5021/ad.2013.25.4.410 24371386 PMC3870207

[B98] Park J. M. Jo J. H. Jin H. Ko H. C. Kim M. B. Kim J. M. (2016). Change in antimicrobial susceptibility of skin-colonizing *Staphylococcus aureus* in Korean patients with atopic dermatitis during ten-year period. *Ann. Dermatol.* 28 470–478. 10.5021/ad.2016.28.4.470 27489430 PMC4969477

[B99] Pascolini C. Sinagra J. Pecetta S. Bordignon V. De Santis A. Cilli L. (2011). Molecular and immunological characterization of *Staphylococcus aureus* in pediatric atopic dermatitis: implications for prophylaxis and clinical management. *Clin. Dev. Immunol.* 2011 1–7. 10.1155/2011/718708 22110527 PMC3205653

[B100] Patel D. Jahnke M. N. (2015). Serious complications from *Staphylococcal aureus* in atopic dermatitis. *Pediatr. Dermatol.* 32 792–796. 10.1111/pde.12665 26337792

[B101] Patel G. K. Wyatt H. Kubiak E. M. Clark S. M. Mills C. M. (2001). Staphylococcus aureus colonization of children with atopic eczema and their parents [2]. *Acta Derm. Venereol.* 81 366–367. 10.1080/000155501317140124 11800148

[B102] Pérez-Laguna V. García-Luque I. Ballesta S. Pérez-Artiaga L. Lampaya-Pérez V. Samper S. (2018). Antimicrobial photodynamic activity of Rose Bengal, alone or in combination with gentamicin, against planktonic and biofilm Staphylococcus aureus. *Photodiagn. Photodyn. Ther.* 21 211–216. 10.1016/j.pdpdt.2017.11.012 29196246

[B103] Pérez-Laguna V. Pérez-Artiaga L. Lampaya-Pérez V. García-Luque I. Ballesta S. Nonell S. (2017). Bactericidal effect of photodynamic therapy, alone or in combination with mupirocin or linezolid, on *Staphylococcus aureus*. *Front. Microbiol.* 8:1002. 10.3389/fmicb.2017.01002 28626456 PMC5454219

[B104] Pinchuk I. V. Beswick E. J. Reyes V. E. (2010). Staphylococcal enterotoxins. *Toxins* 2 2177–2197. 10.3390/toxins2082177 22069679 PMC3153290

[B105] Pozzi G. Asero R. (2010). Skin photodynamic therapy in severe localized atopic dermatitis: a case report. *Br. J. Dermatol.* 163 430–431. 10.1111/j.1365-2133.2010.09823.x 20426774

[B106] Proksch E. Brandner J. M. Jensen J. M. (2008). The skin: an indispensable barrier. *Exp. Dermatol.* 17 1063–1072. 10.1111/j.1600-0625.2008.00786.x 19043850

[B107] Queirós C. Garrido P. M. Maia Silva J. Filipe P. (2020). Photodynamic therapy in dermatology: beyond current indications. *Dermatol. Ther.* [Epub ahead of print] 10.1111/dth.13997 32654315

[B108] Rangel S. M. Paller A. S. (2018). Bacterial colonization, overgrowth, and superinfection in atopic dermatitis. *Clin. Dermatol.* 36 641–647. 10.1016/j.clindermatol.2018.05.005 30217276

[B109] Rawlings A. V. Scott I. R. Harding C. R. Bowser P. A. (1994). Stratum corneum moisturization at the molecular level. *J. Invest. Dermatol.* 124 1099–1110. 10.1111/1523-1747.ep12398620 7963664

[B110] Ring J. Abeck D. Neuber K. (1992). Atopic eczema: role of microorganisms on the skin surface. *Allergy* 47 265–269. 10.1111/j.1398-9995.1992.tb02051.x 1443443

[B111] Rodenbeck D. L. Silverberg J. I. Silverberg N. B. (2016). Phototherapy for atopic dermatitis. *Clin. Dermatol.* 34 607–613. 10.1016/j.clindermatol.2016.05.011 27638440

[B112] Rojo A. Aguinaga A. Monecke S. Yuste J. R. Gastaminza G. España A. (2014). Staphylococcus aureus genomic pattern and atopic dermatitis: may factors other than superantigens be involved? *Eur. J. Clin. Microbiol. Infect. Dis.* 33 651–658. 10.1007/s10096-013-2000-z 24162256

[B113] Roll A. Cozzio A. Fischer B. Schmid-Grendelmeier P. (2004). Microbial colonization and atopic dermatitis. *Curr. Opin. Allergy Clin. Immunol.* 4 373–378. 10.1097/00130832-200410000-00008 15349036

[B114] Rustin M. H. A. (2007). The safety of tacrolimus ointment for the treatment of atopic dermatitis: a review. *Br. J. Dermatol.* 157 861–873. 10.1111/j.1365-2133.2007.08177.x 17854353

[B115] Saravolatz L. D. Markowitz N. Arking L. Pohlod D. Fisher E. (1982). Methicillin-resistant *Staphylococcus aureus*. epidemiologic observations during a community-acquired outbreak. *Ann. Intern. Med.* 96 11–16. 10.7326/0003-4819-96-1-11 7053683

[B116] Schlievert P. M. Case L. C. Strandberg K. L. Abrams B. B. Leung D. Y. M. (2008). Superantigen profile of *Staphylococcus aureus* isolates from patients with steroid-resistant atopic dermatitis. *Clin. Infect. Dis.* 46 1562–1567. 10.1086/586746 18419342 PMC2637450

[B117] Schlievert P. M. Gourronc F. A. Leung D. Y. M. Klingelhutz A. J. (2020). Human keratinocyte response to superantigens. *mSphere* 5:e00803-20. 10.1128/mSphere.00803-20 PMC7568652 33028686

[B118] Schwartz D. M. Kanno Y. Villarino A. Ward M. Gadina M. O’Shea J. J. (2017). JAK inhibition as a therapeutic strategy for immune and inflammatory diseases. *Nat. Rev. Drug Discov.* 16 843–862. 10.1038/nrd.2017.201 29104284

[B119] Silverberg J. I. (2017). Public health burden and epidemiology of atopic dermatitis. *Dermatol. Clin.* 35 283–289. 10.1016/j.det.2017.02.002 28577797

[B120] Simpson E. L. Bieber T. Guttman-Yassky E. Beck L. A. Blauvelt A. Cork M. J. (2016). Two phase 3 trials of dupilumab versus placebo in atopic dermatitis. *N. Engl. J. Med.* 375 2335–2348. 10.1056/nejmoa1610020 27690741

[B121] Simpson E. L. Lacour J. P. Spelman L. Galimberti R. Eichenfield L. F. Bissonnette R. (2020a). Baricitinib in patients with moderate-to-severe atopic dermatitis and inadequate response to topical corticosteroids: results from two randomized monotherapy phase III trials. *Br. J. Dermatol.* 183 242–255. 10.1111/bjd.18898 31995838

[B122] Simpson E. L. Sinclair R. Forman S. Wollenberg A. Aschoff R. Cork M. (2020b). Efficacy and safety of abrocitinib in adults and adolescents with moderate-to-severe atopic dermatitis (JADE MONO-1): a multicentre, double-blind, randomised, placebo-controlled, phase 3 trial. *Lancet* 396 255–266. 10.1016/S0140-6736(20)30732-7 32711801

[B123] Soares J. Lopes C. Tavaria F. Delgado L. Pintado M. (2013). A diversity profile from the staphylococcal community on atopic dermatitis skin: a molecular approach. *J. Appl. Microbiol.* 115 1411–1419. 10.1111/jam.12296 23910049

[B124] Spaulding A. R. Salgado-Pabón W. Kohler P. L. Horswill A. R. Leung D. Y. M. Schlievert P. M. (2013). Staphylococcal and streptococcal superantigen exotoxins. *Clin. Microbiol. Rev.* 26 422–447. 10.1128/CMR.00104-12 23824366 PMC3719495

[B125] Spaulding A. R. Satterwhite E. A. Lin Y. C. Chuang-Smith O. N. Frank K. L. Merriman J. A. (2012). Comparison of *Staphylococcus aureus* strains for ability to cause infective endocarditis and lethal sepsis in rabbits. *Front. Cell. Infect. Microbiol.* 2:18. 10.3389/fcimb.2012.00018 22919610 PMC3417574

[B126] Suh L. Coffin S. Leckerman K. H. Gelfand J. M. Honig P. J. Yan A. C. (2008). Methicillin-Resistant *Staphylococcus aureus* colonization in children with atopic dermaitis. *Pediatr. Dermatol.* 25 528–534. 10.1111/j.1525-1470.2008.00768.x 18950393

[B127] Sule O. Brown N. M. Willocks L. J. Day J. Shankar S. Palmer C. R. (2007). Fusidic acid-resistant *Staphylococcus aureus* (FRSA) carriage in patients with atopic eczema and pattern of prior topical fusidic acid use. *Int. J. Antimicrob. Agents* 30 78–82. 10.1016/j.ijantimicag.2007.02.015 17475448

[B128] Syed A. K. Reed T. J. Clark K. L. Boles B. R. Kahlenberg J. M. (2015). *Staphlyococcus aureus* phenol-soluble modulins stimulate the release of proinflammatory cytokines from keratinocytes and are required for induction of skin inflammation. *Infect. Immun.* 83 3428–3437. 10.1128/IAI.00401-15 26077761 PMC4534673

[B129] Taskapan M. O. Kumar P. (2000). Role of staphylococcal superantigens in atopic dermatitis: from colonization to inflammation. *Ann. Allergy Asthma Immunol.* 84 3–12. 10.1016/S1081-1206(10)62731-7 10674558

[B130] Tomi N. S. Kränke B. Aberer E. (2005). Staphylococcal toxins in patients with psoriasis, atopic dermatitis, and erythroderma, and in healthy control subjects. *J. Am. Acad. Dermatol.* 53 67–72. 10.1016/j.jaad.2005.02.034 15965423

[B131] Totté J. E. E. van der Feltz W. T. Hennekam M. van Belkum A. van Zuuren E. J. Pasmans S. G. M. A. (2016). Prevalence and odds of *Staphylococcus aureus* carriage in atopic dermatitis: a systematic review and meta-analysis. *Br. J. Dermatol.* 175 687–695. 10.1111/bjd.14566 26994362

[B132] Tubby S. Wilson M. Nair S. P. (2009). Inactivation of staphylococcal virulence factors using a light-activated antimicrobial agent. *BMC Microbiol.* 9:211. 10.1186/1471-2180-9-211 19804627 PMC2762988

[B133] Valour F. Tasse J. Trouillet-Assant S. Rasigade J. P. Lamy B. Chanard E. (2014). Methicillin-susceptible *Staphylococcus aureus* clonal complex 398: high prevalence and geographical heterogeneity in bone and joint infection and nasal carriage. *Clin. Microbiol. Infect.* 20 O772–O775. 10.1111/1469-0691.12567 24461054

[B134] van Cleef B. A. G. L. Monnet D. L. Voss A. Krziwanek K. Allerberger F. Struelens M. (2011). Livestockassociated methicillin- resistant *Staphylococcus aureus* in humans. *Europe Emerg. Infect. Dis.* 17 502–505. 10.3201/eid1703.101036 21392444 PMC3166010

[B135] Wainwright M. Maisch T. Nonell S. Plaetzer K. Almeida A. Tegos G. P. (2017). Photoantimicrobials—are we afraid of the light? *Lancet Infect. Dis.* 17 e49–e55. 10.1016/S1473-3099(16)30268-7 27884621 PMC5280084

[B136] Weidinger S. Baurecht H. Schmitt J. (2017). A 5-year randomized trial on the safety and efficacy of pimecrolimus in atopic dermatitis: a critical appraisal. *Br. J. Dermatol.* 177 999–1003. 10.1111/bjd.15827 28868633

[B137] Woo T. E. Kuzel P. (2019). Crisaborole 2% Ointment (Eucrisa) for atopic dermatitis. *Skin Ther. Lett.* 24 4–6. 30970204

[B138] Worm M. Simpson E. L. Thaçi D. Bissonnette R. Lacour J.-P. Beissert S. (2020). Efficacy and safety of multiple dupilumab dose regimens after initial successful treatment in patients with atopic dermatitis. *JAMA Dermatol.* 156:131. 10.1001/jamadermatol.2019.3617 31876900 PMC6990756

[B139] Yagi S. Wakaki N. Ikeda N. Takagi Y. Uchida H. Kato Y. (2004). Presence of staphylococcal exfoliative toxin A in sera of patients with atopic dermatitis. *Clin. Exp. Allergy* 34 984–993. 10.1111/j.1365-2222.2004.1687.x 15196290

[B140] Yarwood J. M. Leung D. Y. M. Schlievert P. M. (2000). Evidence for the involvement of bacterial superantigens in psoriasis, atopic dermatitis, and Kawasaki syndrome. *FEMS Microbiol. Lett.* 192 1–7. 10.1016/S0378-1097(00)00400-6 11040420

[B141] Yeung M. Balma-Mena A. Shear N. Simor A. Pope E. Walsh S. (2011). Identification of major clonal complexes and toxin producing strains among Staphylococcus aureus associated with atopic dermatitis. *Microbes Infect.* 13 189–197. 10.1016/j.micinf.2010.10.023 21093604

[B142] Yu J. Luo Y. Zhu Z. Zhou Y. Sun L. Gao J. (2019). A tryptophan metabolite of the skin microbiota attenuates inflammation in patients with atopic dermatitis through the aryl hydrocarbon receptor. *J. Allergy Clin. Immunol.* 143 2108.e12–2119.e12. 10.1016/j.jaci.2018.11.036 30578876

[B143] Zollner T. M. Wichelhaus T. A. Hartung A. Von Mallinckrodt C. Wagner T. O. F. Brade V. (2000). Colonization with superantigen-producing *Staphylococcus aureus* is associated with increased severity of atopic dermatitis. *Clin. Exp. Allergy* 30 994–1000. 10.1046/j.1365-2222.2000.00848.x 10848922

[B144] Zuuren E. J. Fedorowicz Z. Arents B. W. M. (2017). Emollients and moisturizers for eczema: abridged cochrane systematic review including *GRADE* assessments. *Br. J. Dermatol.* 177 1256–1271. 10.1111/bjd.15602 28432721

